# Chlorogenic acid attenuates TBI-induced neuroinflammation by suppressing cGAS–STING signaling

**DOI:** 10.1016/j.neurot.2026.e01085

**Published:** 2026-09-28

**Authors:** Xu Yang, Kun He, Yulian Zhang, Hanhan Dang, Zixi Wang, Yunsheng Zhang, Chuanpeng Zhang, Pengyu Chen, Tongbo Zhang, Li Zhang, Yanbing Yu

**Affiliations:** a Peking University China-Japan Friendship School of Clinical Medicine, Beijing, China; b Department of Neurosurgery, China-Japan Friendship Hospital, Beijing, China; c Department of Pain Medicine, China-Japan Friendship Hospital, Beijing, China; d China-Japan Friendship Hospital (Institute of Clinical Medical Sciences), Chinese Academy of Medical Sciences & Peking Union Medical College, Beijing, China; e Department of Neurosurgery, Civil Aviation General Hospital, Beijing, China; f Department of Neurology, Johns Hopkins School of Medicine, Baltimore, USA

**Keywords:** Traumatic brain injury, Chlorogenic acid, cGAS–STING signaling, Microglia, Neuroinflammation

## Abstract

Traumatic brain injury (TBI) induces persistent neuroinflammation that contributes to secondary brain injury and neurological dysfunction. The cyclic GMP-AMP synthase–stimulator of interferon genes (cGAS–STING) pathway is a key regulator of post-traumatic innate immune responses. Although chlorogenic acid (CGA) possesses well-established anti-inflammatory properties, the mechanisms underlying its protective effects after TBI remain incompletely understood. CGA efficacy was evaluated using a murine controlled cortical impact model. Bulk and single-cell RNA sequencing, network pharmacology, and mouse-specific molecular docking simulations were combined to identify candidate pathways. The underlying mechanism was further investigated *in vivo* and in LPS-stimulated BV2 microglia using the STING agonist DMXAA. CGA treatment significantly improved neurological function, enhanced cognitive performance, and alleviated blood-brain barrier disruption after TBI. Integrated analyses implicated cGAS–STING signaling as a candidate pathway associated with CGA action, with single-cell transcriptomic analysis showing relatively high pathway activity in microglia and infiltrating myeloid populations. Mechanistically, CGA reduced cGAS expression, inhibited the phosphorylation of STING, TBK1, and IRF3, and decreased pro-inflammatory cytokine production *in vivo*. *In vitro*, CGA decreased the expression of inflammatory and interferon-stimulated genes in a concentration-dependent manner and reduced reactive oxygen species accumulation; these effects were largely reversed by DMXAA. CGA attenuates neuroinflammatory responses and improves neurological outcomes after TBI, with early suppression of cGAS–STING signaling potentially contributing to these effects. These findings support further investigation of cGAS–STING signaling as a potential therapeutic target in TBI.

## Graphical abstract

**Figure:**
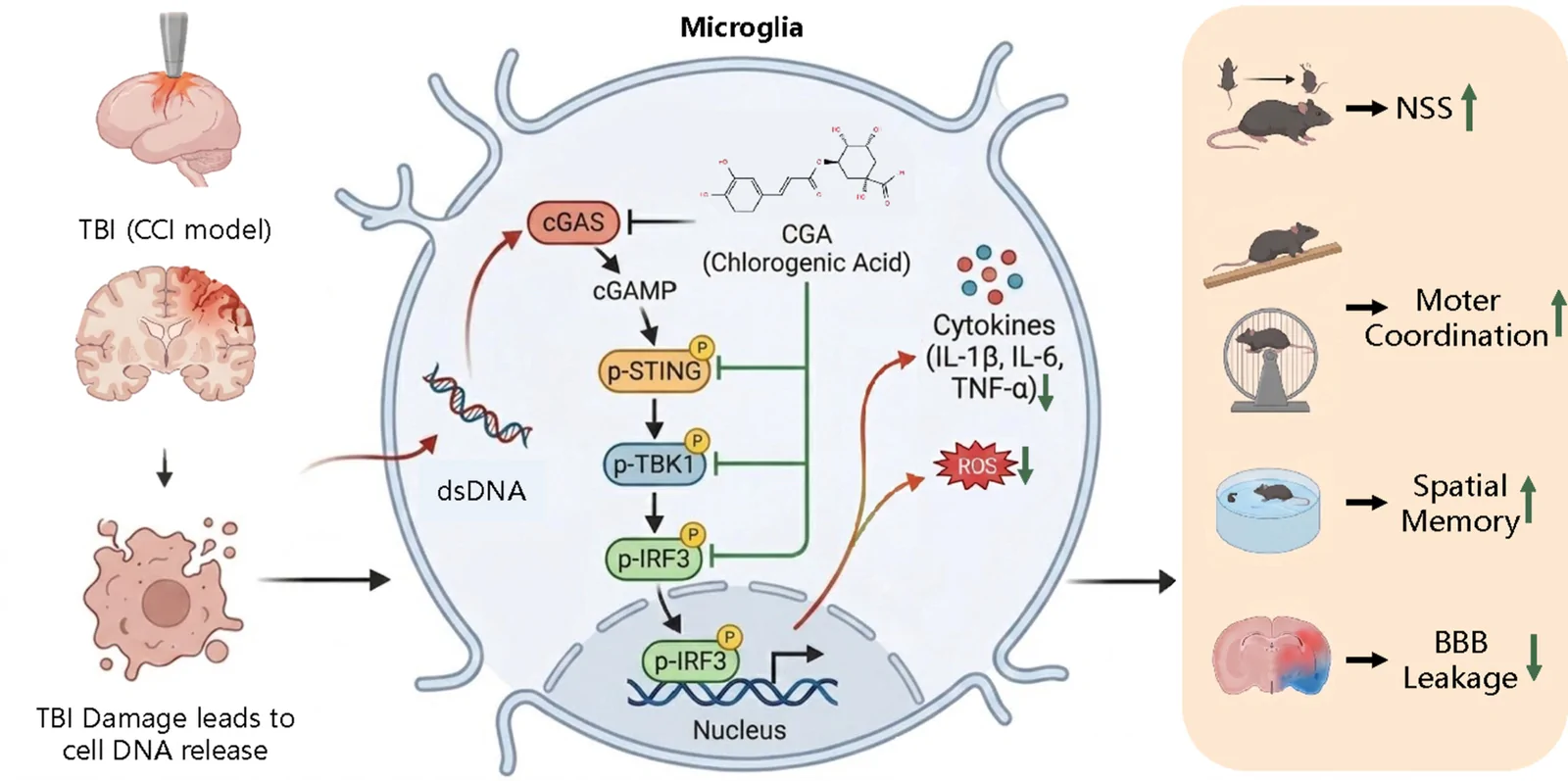

## Introduction

Traumatic brain injury (TBI) continues to impose a substantial global health burden because of its high mortality and the persistent neurological deficits experienced by many survivors [[1], [2], [3]]. Although the immediate mechanical damage is largely irreversible, the subsequent progression of injury is characterized by a series of evolving biological responses that extend well beyond the initial trauma [4]. Among these secondary pathological processes, sustained neuroinflammation is closely associated with cerebral edema, blood-brain barrier (BBB) disruption, neuronal loss, and progressive neurological dysfunction [5,6]. Microglia, the resident immune cells of the central nervous system (CNS), rapidly respond to tissue damage and regulate the post-traumatic inflammatory milieu [7]. However, persistent activation of these cells can maintain excessive production of inflammatory mediators and reactive oxygen species (ROS), thereby amplifying secondary brain injury rather than supporting tissue repair [8,9]. Accordingly, strategies aimed at restoring microglial immune homeostasis have become an important direction for developing disease-modifying therapies after TBI.

The cyclic GMP–AMP synthase (cGAS)–stimulator of interferon genes (STING) pathway is a pivotal component of the innate immune system that links cytosolic DNA sensing to inflammatory signaling [[10], [11], [12], [13]]. Following traumatic injury, mitochondrial dysfunction, nuclear damage, and cellular stress promote the accumulation of aberrant double-stranded DNA within the cytoplasm, leading to activation of the cGAS–STING signaling cascade [14,15]. Activated STING subsequently initiates downstream signaling through TBK1 and IRF3, promoting the production of type I interferons and other inflammatory mediators that contribute to secondary tissue injury. Increasing evidence indicates that excessive activation of this pathway is involved in the progression of multiple central nervous system disorders and that pharmacological inhibition of cGAS–STING signaling can alleviate neuroinflammation and improve neurological recovery [16,17].

Chlorogenic acid (CGA), a plant-derived polyphenolic compound, possesses documented anti-inflammatory and antioxidative properties [18,19]. Although CGA has shown beneficial effects in several neurological disorders, the molecular mechanisms underlying its actions following traumatic brain injury remain largely undefined [20,21]. In the present study, we hypothesized that inhibition of microglial cGAS–STING signaling contributes to the anti-inflammatory effects of CGA after TBI. To test this hypothesis, we combined complementary *in vivo* and *in vitro* experiments with transcriptomic analyses, network pharmacology, molecular docking based on mouse-derived protein structures, and single-cell transcriptomic profiling, followed by experimental validation.

## Methods

### Animals and ethical statement

Male C57BL/6 mice (7 weeks of age, 20–25 g; SPF Biotechnology Co., Ltd., Beijing, China) were housed under specific pathogen-free conditions with controlled temperature (23 ± 2 °C), relative humidity (45–60%), and a 12-h light/dark cycle. Animals had ad libitum access to standard chow and water and were acclimated for 7 days before experimentation. All animal procedures were approved by the Institutional Animal Welfare and Ethics Committee of China-Japan Friendship Hospital (Approval No. ZRDWLL240035). Animal husbandry and care complied with the ARRIVE Guidelines 2.0 [22] and the Guide for the Care and Use of Laboratory Animals (NIH). Upon reaching experimental endpoints, mice were deeply anesthetized with sodium pentobarbital (50 mg/kg, i.p.) and euthanized by cervical dislocation.

### TBI model induction and administration

Experimental TBI was established using a controlled cortical impact (CCI) model as previously described [23,24]. Anesthesia was induced with 3% isoflurane and maintained at 2% throughout surgery while each mouse was secured in a digital stereotaxic framework (RWD Life Science, Shenzhen, China). Following a midline scalp incision, a craniotomy was performed over the left sensorimotor cortex with the dura mater left intact. Cortical injury was induced using a pneumatic impact device (68099II, RWD Life Science) equipped with a 3-mm impactor tip at a velocity of 3.5 m/s, an impact depth of 2 mm, and a dwell time of 500 ms. The coordinates for the injury epicenter were situated at −2.00 mm anterior-posterior (A/P) and −2.50 mm medial-lateral (M/L) relative to bregma. Core body temperature was kept constant at 37.5 ± 0.5 °C using a heating system (Model 69001, RWD Life Science). Sham controls underwent identical anesthetic and skull opening steps without the pneumatic tissue impact.

Chlorogenic acid (CGA; purity 99.53%; MCE, HY-N0055) was dissolved in sterile saline and administered via the tail vein at 2 h, 1 d, and 2 d after injury. Mice received either 50 mg/kg (lCGA) or 150 mg/kg (hCGA) CGA. The selected dose range was informed by previous *in vivo* studies demonstrating biological activity of CGA at doses within approximately 50–150 mg/kg, including dose-ranging studies using 50, 100, and 150 mg/kg CGA. The initial 2-h treatment time point was selected to target the early secondary injury phase and was also supported by previous neuroprotection studies in which CGA administered 2 h after experimental cerebral ischemia produced beneficial effects [25,26]. Repeated administration at 1 and 2 d was used to maintain CGA exposure during the early post-traumatic inflammatory period, preceding the prominent cGAS–STING activation observed at 3 dpi in our temporal transcriptomic analysis. An additional 100 mg/kg group was included only during the preliminary dose-screening stage to evaluate concentration-dependent molecular responses. The overall experimental workflow is summarized in Supplementary Fig. 1.

### Neurobehavioral assessments

Behavioral assessments were performed by two investigators blinded to treatment allocation. Before each behavioral assessment, mice were acclimated to the testing environment for 2 h under identical conditions.

● **Neurological Severity Score (NSS):** Neurological deficits were assessed at 3 days post-injury (dpi) using a standard 10-point NSS system that evaluates motor function, sensory responses, balance, and reflexes [27].
● **Beam Balance Test:** Motor coordination and balance were evaluated at 3 dpi. Following two days of pre-training, mice traversed a suspended wooden beam (10 mm in diameter and 100 cm in length) from an illuminated starting platform to a dark goal box. Crossing time was averaged from three consecutive trials.
● **Rotarod Test:** Motor coordination was assessed at 7 dpi using an accelerating rotarod. After adaptation and training sessions, mice were tested under accelerating conditions from 4 to 40 rpm over 5 min. The average latency to fall from two independent trials was used for analysis.
● **Open Field Test:** Exploratory activity and anxiety-like behavior were evaluated at 28 dpi inside a square enclosure (500 × 500 × 350 mm) containing a central zone (300 × 300 mm). Mice were allowed to explore the arena freely for 5 min while movement trajectories were automatically recorded using the LabMaze tracking system. Total distance traveled, mean velocity, time spent in the center, and time spent in the peripheral zone were quantified.
● **Morris Water Maze Test:** Spatial learning and reference memory were evaluated during an acquisition block (22–26 dpi) and a retention probe (27 dpi) using the LabMaze tracking system. The apparatus comprised a circular pool (1.2 m diameter) filled with water (22–24 °C) rendered opaque with a non-toxic titanium dioxide suspension. A hidden escape platform (6 cm diameter) was submerged 1 cm below the surface in a designated target quadrant. During the 5-day acquisition window, mice performed two daily training trials to locate the hidden platform within 60 s. A two-trial-per-day schedule was used to provide repeated spatial acquisition over five consecutive days while limiting excessive swimming-related fatigue and stress in mice recovering from TBI; reduced-trial training paradigms have also been used in experimental TBI studies [28]. Unsuccessful animals were guided to the site and allowed to rest there for 30 s. A probe trial was performed on day 27 after platform removal. Escape latency during acquisition training, together with latency to the target quadrant, time spent in the target quadrant, and swimming speed during the probe trial, were analyzed using the LabMaze tracking system.

### Blood-brain barrier (BBB) permeability assessment

BBB disruption following TBI was evaluated by quantifying Evans Blue (EB) leakage into brain tissue. After intravenous administration of 2% EB solution (4 mL/kg) through the tail vein, mice were maintained for 1 h to allow dye circulation. Animals were then anesthetized and perfused transcardially with 100 mL sterile saline to eliminate intravascular EB. The injured hemisphere was subsequently isolated, weighed, and homogenized in 1 mL extraction buffer containing equal volumes of PBS (0.5 mL) and 50% trichloroacetic acid (0.5 mL). Following overnight incubation at 4 °C, tissue homogenates were centrifuged at 15,000 rpm for 30 min at 4 °C. The EB concentration in the collected supernatant was determined spectrophotometrically at 620 nm based on a standard curve and expressed as micrograms per gram of brain tissue.

### LC–MS/MS analysis of CGA in brain tissue

To determine whether intravenously administered CGA reaches brain tissue after TBI, an additional cohort of TBI mice receiving CGA (150 mg/kg, i.v.) was used for LC–MS/MS analysis. At 30 min after CGA administration, mice were euthanized and the ipsilateral injured and contralateral hemispheres were collected separately (n = 6). Brain samples were weighed, extracted with 0.5 mL methanol, homogenized for 5 min, and centrifuged at 13,000 rpm for 10 min at 4 °C. The supernatants were filtered through 0.22-μm membranes before analysis. CGA was quantified using an UltiMate 3000 RS liquid chromatography system coupled to a Q Exactive high-resolution mass spectrometer (Thermo Fisher Scientific). Chromatographic separation was performed using an Agela Venusil C18 Plus column (50 × 2.1 mm, 5 μm), with 0.1% formic acid in water and methanol as the mobile phases. Mass spectrometric detection was performed in negative electrospray ionization mode using parallel reaction monitoring, with precursor and product ions of m/z 353.0882 and 191.05484, respectively. Quantification was based on a matrix-matched calibration curve prepared using blank brain tissue extract with weighted linear regression (1/x^2^).

### RNA sequencing and bioinformatics analysis

Total RNA was extracted from brain tissue samples using TRIzol reagent (Invitrogen, USA) according to the manufacturer's instructions. RNA concentration and purity were determined using a NanoDrop 2000 spectrophotometer (Thermo Fisher Scientific, USA). RNA sequencing was performed by OE Biotech Co., Ltd. (Shanghai, China).

Differential gene expression analysis was conducted using the DESeq2 package, with genes meeting the criteria of $\left|log_{2}FC\right|>0.585$ and p<0.05 considered differentially expressed genes (DEGs). To identify potential molecular links between CGA and TBI, disease-associated genes were collected from the OMIM and GeneCards databases, whereas putative CGA-related targets were obtained from SwissTargetPrediction, TCMSP, BindingDB, CTD, and ChEMBL databases. These datasets were integrated with transcriptomic DEGs to identify candidate genes shared across datasets.

The interaction network among overlapping genes was constructed using the STRING database [29]. To identify core topological hubs, five separate network metrics (MCC, DMNC, Degree, BottleNeck, and EPC) were calculated using the cytoHubba plugin within the Cytoscape workspace (v3.10.4) [30]. Functional annotation of the identified genes was performed using Gene Ontology (GO) and Kyoto Encyclopedia of Genes and Genomes (KEGG) enrichment analyses using the clusterProfiler package [31]. Data visualization was performed using the GSEABase and ggplot2 packages.

### Molecular docking simulation

To investigate the potential interactions between CGA and key components of the cGAS–STING signaling pathway, molecular docking analysis was performed using mouse-derived protein structures. The three-dimensional crystal structures of murine cGAS (PDB ID: 9J2Z), STING (PDB ID: 4LOL), TBK1 (PDB ID: 4JLC), and IRF3 (PDB ID: 7JFM) were obtained from the RCSB Protein Data Bank. The chemical structure of CGA was retrieved from the PubChem database (CID: 1794427).

Before docking, protein structures and ligand molecules were prepared by adding polar hydrogens and assigning Gasteiger charges. Molecular docking was performed using AutoDock Vina (version 1.5.7) [32,33]. The predicted binding conformations and intermolecular interactions were subsequently visualized using PyMOL (version 3.1.8) and Discovery Studio Visualizer (version 25.1.0.2) to characterize the potential interactions between CGA and the selected target proteins.

### Cellular thermal shift assay (CETSA)

To further evaluate the potential interaction of CGA with cGAS–STING pathway proteins, CETSA was performed using BV2 cells. Samples were treated with CGA at 900 μM or an equivalent volume of DMSO for 2 h. Aliquots were subsequently heated at 37, 40, 43, 46, 49, 52, 55, 58, or 61 °C for 3 min, followed by cooling and protein extraction. Soluble protein fractions were collected and analyzed by western blotting for cGAS, STING, TBK1, and IRF3. Protein band intensities were quantified using ImageJ and normalized to the corresponding signal at 37 °C.

### Single-cell RNA sequencing

Public single-cell RNA sequencing (scRNA-seq) data were obtained from the Gene Expression Omnibus under accession number GSE302863 [34]. The original dataset comprised five scRNA-seq libraries representing cortical tissue from sham mice and injury-site cortical tissue collected at 1, 3, 7, and 14 d after TBI. In the original study, cortical tissues were minced and enzymatically dissociated using 8 U/mL papain and 125 U/mL DNase I, followed by gentle mechanical dissociation. Enzymatic digestion was terminated with 10% ovomucoid, cell suspensions were passed through 70-μm strainers, and tissue debris was removed by 30% Percoll density-gradient centrifugation. Samples with cell viability >85% were used for library preparation. Approximately 5188–11,934 cells per sample were loaded onto the 10 × Genomics Chromium platform, and the resulting libraries were sequenced using an Illumina HiSeq platform. Quality control filtering, library size normalization, and downstream dimension reduction of the cellular expression matrix were managed through the Seurat framework (v4.4.2) [35]. Cells were retained if they met the following quality-control criteria: nFeature_RNA >200, nFeature_RNA <5,000, and nCount_RNA <20,000. Cells outside these thresholds were excluded before global scaling normalization. Highly variable genes (n = 2000) were selected for principal component analysis (PCA), and the first 20 principal components were used for downstream clustering (resolution = 0.6) and t-SNE visualization.

Major neural, immune, vascular, and stromal cell populations were annotated using established canonical marker genes: microglia (*Tmem119*, *P2ry12*, *Cx3cr1*); astrocytes (*Gfap*, *Aqp4*); oligodendrocytes (*Olig1*, *Olig2*, *Mog*, *Mag*); neurons (*Syp*, *Tubb3*, *Gad1*, *Gad2*); T cells (*Cd8a*, *Cd3e*); macrophages (*Ms4a7*, *Ccr2*, *Pf4*); dendritic cells (*Flt3*, *Cd40*, *Cd80*); neutrophils (*Lcn2*, *S100a8*); endothelial cells (*Cldn5*, *Abcb1a*, *Flt1*); mural cells (*Vtn*, *Rgs5*); and choroid plexus epithelial cells (*Ttr*).

To characterize the cellular distribution of cGAS–STING signaling activity, the AUCell algorithm was applied to generate pathway enrichment scores for individual cells based on the expression ranking matrix of core pathway genes, including *Cgas*, *Sting1*, *Tbk1*, and *Irf3*. The resulting cGAS–STING activity scores were projected onto t-SNE embeddings to compare pathway activity among different cell populations.

### Cell culture and treatments

The murine microglial cell line BV2 (SCSP-5208) was obtained from the National Collection of Authenticated Cell Cultures. Cells were cultured in Dulbecco's Modified Eagle Medium (DMEM; Gibco, USA) supplemented with 10% fetal bovine serum (FBS; Gibco, USA) and 1% penicillin-streptomycin solution (Pricella Biotechnology, Wuhan, China) under standard conditions of 37 °C and 5% ${\text{CO}}_{2}$ in a humidified incubator.

To induce a standardized pro-inflammatory microglial phenotype, BV2 cells were stimulated with 1 μg/mL lipopolysaccharide (LPS; Sigma, USA). LPS was selected as a reproducible inflammatory stimulus commonly used in BV2 microglia and was not intended to reproduce the sterile cytosolic DNA-triggered upstream events of TBI. CGA was simultaneously added at the indicated concentrations (300, 600, or 900 μM). For pathway rescue experiments, the STING agonist DMXAA (10 μM; TargetMol, USA) was added to restore STING signaling.

### Intracellular ROS measurement

Intracellular reactive oxygen species (ROS) levels were assessed utilizing a DCFH-DA fluorescent probe (Solarbio, CA1410). After completion of the indicated treatments, BV2 cells were incubated with 2.5 μM DCFH-DA for 15 min at 37 °C in the dark. Fluorescence signals were acquired using a Beckman flow cytometer, and ROS levels were analyzed with FlowJo software (version 10.9.0).

### Quantitative real-time PCR (qRT-PCR)

Total RNA was isolated from BV2 cells using the RNApure Fast Tissue & Cell Kit (Cwbio, CW0599S). Complementary DNA templates were synthesized through reverse transcription using HiFiScript gDNA Removal RT MasterMix (Cwbio, CW2020). Real-time quantitative PCR amplifications were monitored on a QuantStudio 5 system (Thermo Fisher Scientific, USA) utilizing SuperStar Universal SYBR Green Master Mix (Cwbio, CW3360). Relative transcript abundance was evaluated against endogenous *Gapdh* controls and calculated using the comparative threshold method ($2^{-\Delta \Delta C_{t}}$). Primer sequences used in this study are listed in Supplementary Table 1.

### Protein extraction and western blotting

Protein samples were prepared from pericontusional brain tissues and BV2 cells using RIPA lysis buffer (Beyotime Biotechnology, Shanghai, China) supplemented with protease inhibitors (MCE, HY-K0010) and phosphatase inhibitors (MCE, HY-K0022 and HY-K0023). Protein concentrations were determined using a BCA Protein Assay Kit (Proteintech, Wuhan, China).

Equal amounts of denatured samples were resolved using sodium dodecyl sulfate-polyacrylamide gel electrophoresis (SDS-PAGE) (Beyotime Biotechnology) and transferred onto polyvinylidene fluoride (PVDF) membranes (Millipore, Boston, USA). After 1 h of blocking at room temperature using 5% BSA buffer (Solarbio, A88020), membranes underwent overnight incubation at 4 °C with primary antibodies against cGAS (1:1000, CST, 79978), p-STING (1:1000, CST, 72971), STING (1:1000, CST, 13647), *p*-TBK1 (1:1000, CST, 5483), TBK1 (1:1000, Selleck, F0525), *p*-IRF3 (1:1000, CST, 29047), IRF3 (1:1000, PTG, 66670-1-Ig), ZO-1 (1:5000, PTG, 21773-1-AP), Occludin (1:1000, Selleck, F22C23), Claudin-5 (1:1000, Selleck, K7A2), GAPDH (1:1000, PTG, HRP-60004), and Tubulin (1:1000, PTG, HRP-66031). Following incubation with the corresponding horseradish peroxidase (HRP)-conjugated secondary antibodies, protein bands were visualized using Immobilon Western Chemiluminescent HRP Substrate (Abbkine, Wuhan, China). Protein expression levels were quantified using ImageJ software (version 1.54f).

### Immunofluorescence (IF) staining

Brain sections (5 μm thickness) were subjected to tissue permeabilization and blocking procedures, followed by a 4 °C overnight incubation with primary antibodies targeting IBA-1 (1:100, Abcam, ab178846), TMEM119 (1:250, PTG, 27585-1-AP), cGAS (1:100, PTG, 29958-1-AP), *p*-IRF3 (1:100, CST, 29047), and p-STING (1:100, Invitrogen, PA5-105674). After thorough rinsing, sections were incubated with the corresponding species-specific fluorophore-conjugated secondary antibodies (Abcam), and nuclei were counterstained with DAPI. Fluorescence images were acquired using a laser scanning confocal microscope (Axio Imager 2, Carl Zeiss, Oberkochen, Germany). For quantitative analysis, the mean fluorescence intensities of cGAS, p-STING, and *p*-IRF3 within IBA-1-positive cells were measured using ImageJ software (version 1.54f) under identical image-acquisition settings across experimental groups.

### Enzyme-linked immunosorbent assay (ELISA)

Brain tissue concentrations of inflammatory cytokines were quantified using ELISA. Concentrations of IL-1β and IL-6 in brain homogenates were measured using commercial ELISA kits (Solarbio, SEKM-0002 and SEKM-0007) according to the manufacturer's instructions.

### Statistical analysis

All statistical analyses were performed using GraphPad Prism software (version 10.1.2). Data are presented as the mean ± standard deviation (SD). The Shapiro–Wilk test was used to assess normality. Comparisons among multiple groups were performed using one-way analysis of variance (ANOVA) followed by Dunnett's multiple comparisons test, unless otherwise specified. CGA concentrations in paired ipsilateral and contralateral brain tissues from the same mice were compared using a two-tailed paired *t*-test. For the *in vitro* rescue experiments, one-way ANOVA followed by Sidak's multiple comparisons test was used. Morris water maze acquisition data were analyzed using two-way repeated-measures ANOVA followed by Sidak's multiple comparisons test. CETSA thermal stability curves were analyzed using two-way repeated-measures ANOVA with Geisser–Greenhouse correction, followed by Sidak's multiple comparisons test. A two-sided *P* < 0.05 was considered statistically significant.

## Results

### CGA improves neurological recovery and preserves BBB integrity after TBI

To evaluate the therapeutic effects of CGA after TBI, mice subjected to CCI underwent a series of behavioral and histological assessments. Neurological deficits were first evaluated using the neurological severity score (NSS). Compared with sham-operated mice, TBI animals exhibited significantly increased NSS scores. High-dose CGA significantly reduced NSS scores compared with vehicle-treated TBI mice (adjusted *P* = 0.0002), whereas the effect of low-dose CGA did not reach statistical significance (adjusted *P* = 0.0812; Fig. 1A). Motor coordination and balance were further examined using the beam balance and rotarod tests. Following TBI, mice exhibited prolonged beam traversal time, reduced rotarod latency, and decreased rotarod distance (Fig. 1B–D). Both low- and high-dose CGA shortened beam crossing time (Fig. 1B), whereas significant improvements in rotarod latency and distance were observed with high-dose CGA but not with low-dose CGA (Fig. 1C and D).

**Fig. 1:**
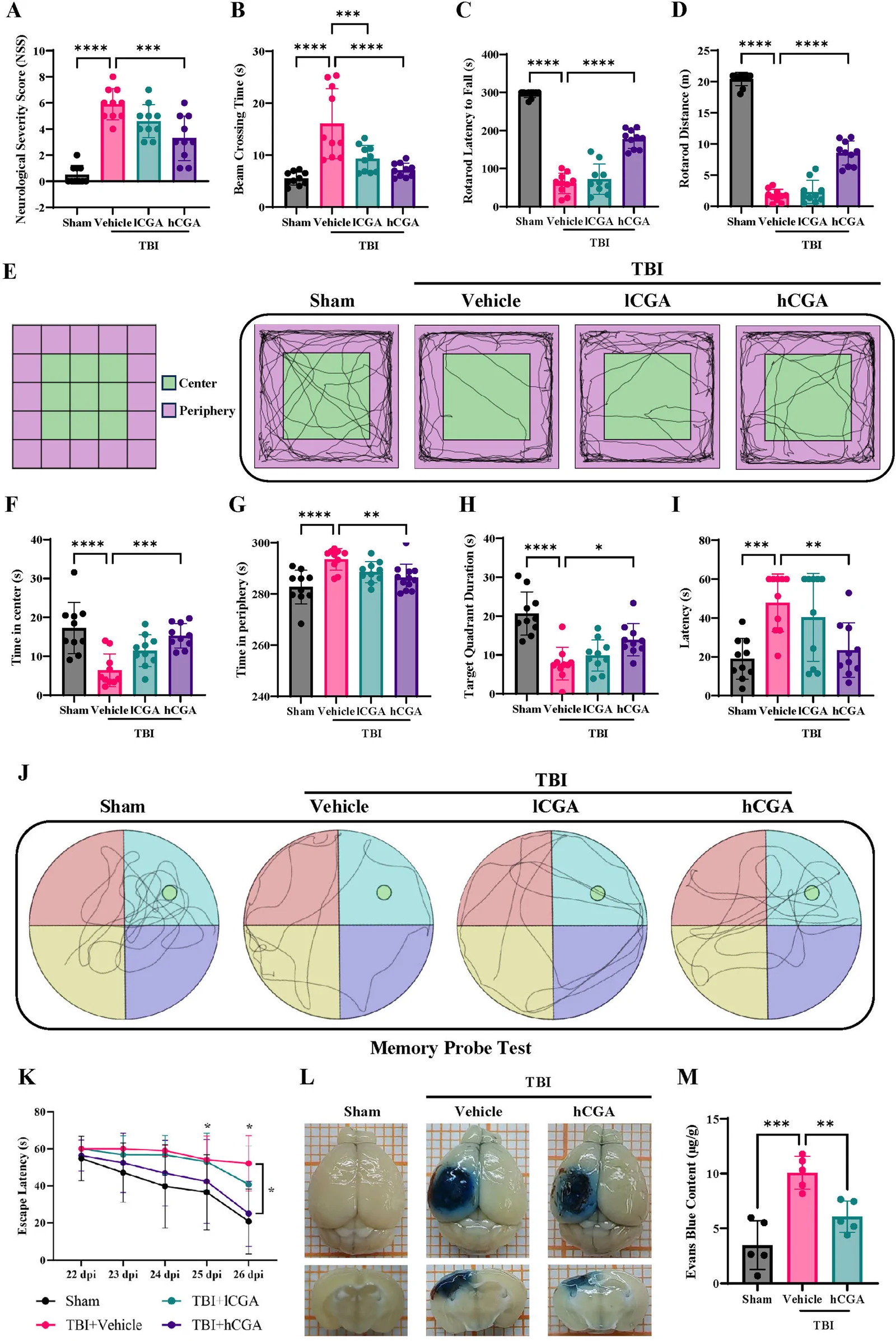
CGA alleviates TBI-induced motor deficits and anxiety-like behaviors, improves spatial learning and memory, and preserves blood-brain barrier integrity after TBI. (A) Neurological Severity Score (NSS) at 3 dpi. (B) Beam crossing time in the beam balance test at 3 dpi. (C) Latency to fall in the accelerating rotarod test at 7 dpi. (D) Total distance traveled in the accelerating rotarod test at 7 dpi. (E) Representative tracking trajectories in the open-field test. Quantitative analysis of (F) time spent in the center zone, (G) time spent in the periphery. Quantitative assessment of spatial memory retention showing (H) target quadrant duration, (I) latency to find the target platform location. (J) Representative swimming trajectories during the Morris water maze probe trial. (K) Escape latency across the 5-day spatial acquisition training phase (22–26 dpi). (L) Representative images of Evans Blue extravasation in the injured hemispheres. (M) Spectrophotometric quantification of Evans Blue content in cortical tissues. Data are presented as mean ± SD. For behavioral tests (A–K), n = 10 mice per group; for Evans Blue extravasation (L, M), n = 3 mice per group. Statistical comparisons were performed using one-way ANOVA followed by Dunnett's multiple comparisons test, except for the Morris water maze acquisition data (K), which were analyzed using two-way repeated-measures ANOVA followed by Sidak's multiple comparisons test. ns, not significant; ∗*P* < 0.05, ∗∗*P* < 0.01, ∗∗∗*P* < 0.001, and ∗∗∗∗*P* < 0.0001.

Exploratory activity and anxiety-like behavior were further assessed using the open-field test. TBI mice spent less time in the center area and more time in the peripheral zone (Fig. 1E–G), whereas CGA treatment partially restored these behavioral abnormalities. No significant differences were observed in total distance traveled or mean velocity among experimental groups (Supplementary Fig. 2A and B), indicating that these behavioral improvements were unlikely to result from altered locomotor activity.

Spatial learning and memory were assessed using the Morris water maze during the chronic phase after injury. During the acquisition period, CGA-treated mice showed progressively shorter escape latencies compared with vehicle-treated TBI mice (Fig. 1K). During the probe trial, high-dose CGA-treated mice spent more time in the target quadrant (adjusted *P* = 0.0123) and reached the target location more rapidly (adjusted *P* = 0.0048) than vehicle-treated TBI mice (Fig. 1H and I). Although TBI mice showed reduced swimming distance and speed during the probe trial, neither low- nor high-dose CGA significantly altered these parameters compared with vehicle-treated TBI mice (Supplementary Fig. 2C and D). Together with the comparable locomotor activity observed in the open-field test, these findings indicate that the improved Morris water maze performance was unlikely to result from a generalized enhancement of locomotor performance. To assess whether CGA attenuated BBB disruption after TBI, Evans Blue extravasation was measured. High-dose CGA treatment significantly reduced EB leakage into the injured brain tissue compared with vehicle-treated TBI mice (adjusted *P* = 0.0065; Fig. 1L and M), indicating improved BBB integrity after injury. To further examine the structural basis of the improved BBB integrity, the expression of the tight-junction proteins ZO-1, Occludin, and Claudin-5 was assessed by western blotting. TBI reduced the expression of these tight-junction proteins, whereas high-dose CGA treatment restored their expression toward sham levels (Supplementary Fig. 2E–H). These findings suggest that preservation of tight-junction proteins may contribute to the reduced BBB permeability observed after CGA treatment.

### Transcriptomic and network-based analyses identify cGAS–STING signaling as a candidate pathway associated with CGA action

To explore the molecular mechanisms underlying CGA-mediated neuroprotection after TBI, we integrated transcriptomic profiling with network pharmacology analysis. Candidate genes were obtained by intersecting CGA-associated targets, TBI-related genes, and differentially expressed genes identified from the temporal transcriptomic dataset (Fig. 2A). This integrative analysis identified 103 overlapping candidate genes. KEGG pathway enrichment analysis was subsequently performed using the overlapping genes, which revealed significant enrichment of inflammation-related signaling pathways (Fig. 2C). To further prioritize biologically relevant candidates and reduce potential bias from individual network algorithms, five complementary topological algorithms (MCC, DMNC, Degree, BottleNeck, and EPC) were applied to the protein–protein interaction (PPI) network. Nine consensus hub genes were identified and used for subsequent network construction and analysis (Fig. 2B–D). Functional enrichment analysis of these hub genes demonstrated a close association with innate immune and inflammatory signaling pathways, among which the cGAS–STING signaling pathway showed prominent enrichment in both KEGG and GO analyses (Fig. 2F and G). These findings identified cGAS–STING signaling as a candidate pathway associated with the protective effects of CGA following TBI.

**Fig. 2:**
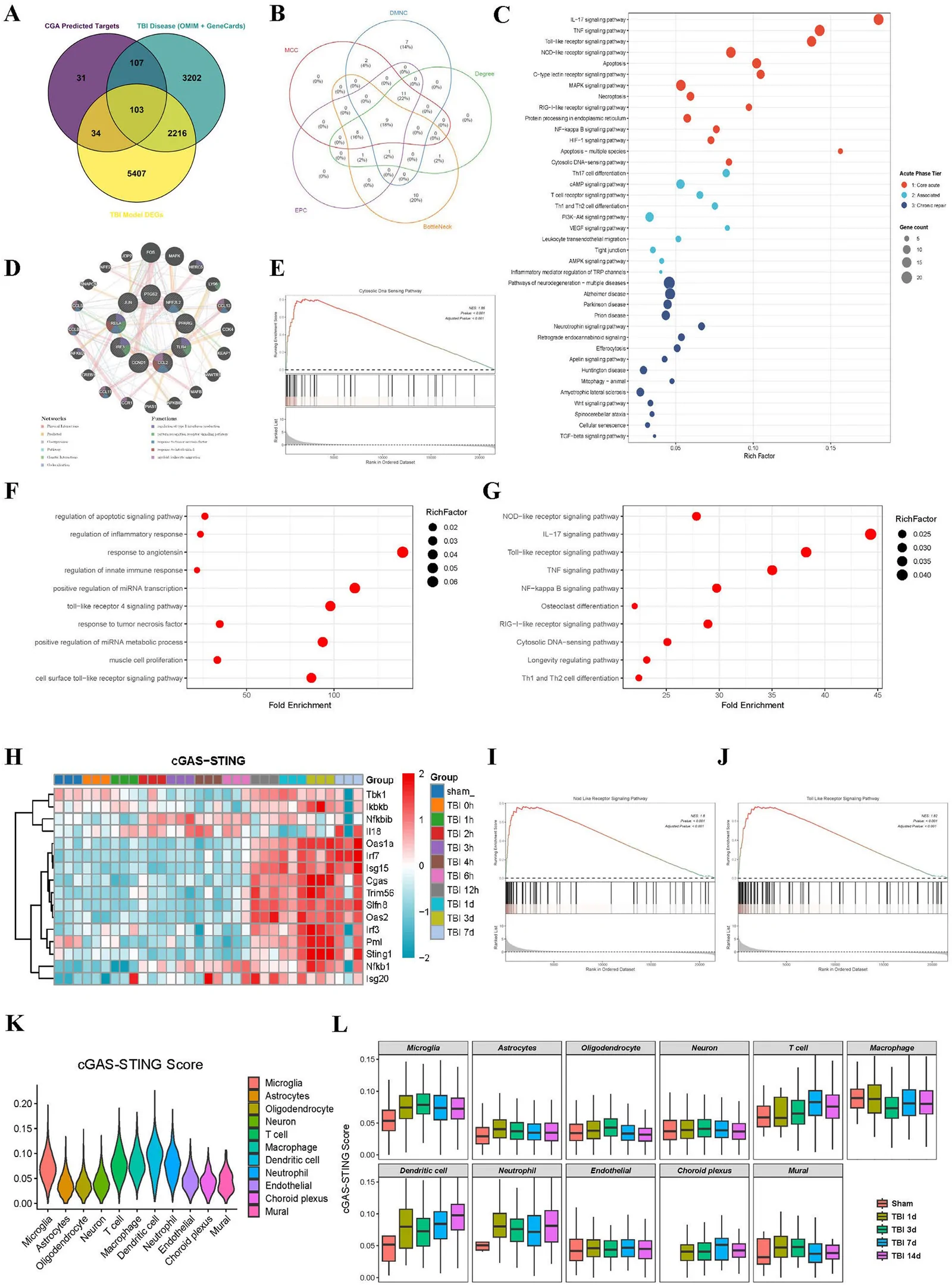
Integrated transcriptomic, network-based, and single-cell analyses identify cGAS–STING signaling as a candidate pathway associated with CGA action after TBI. (A) Venn diagram showing the overlap among predicted CGA targets, TBI-associated genes, and differentially expressed genes identified in the TBI transcriptomic dataset. (B) Identification of consensus hub genes using five cytoHubba topological algorithms (MCC, DMNC, Degree, BottleNeck, and EPC). (C) KEGG pathway enrichment analysis of the overlapping candidate genes. (D) Protein–protein interaction network of the overlapping genes. (E) GSEA of the cytosolic DNA-sensing pathway after TBI. (F, G) GO and KEGG enrichment analyses of the consensus hub genes. (H) Heatmap showing the temporal expression profiles of cGAS–STING-related genes after TBI. (I, J) GSEA plots of NOD-like receptor and Toll-like receptor signaling pathways after TBI. (K) Violin plot showing the distribution of cGAS–STING activity scores across major CNS cell populations. (L) Temporal changes in cGAS–STING activity scores within individual cell populations from sham to 1, 3, 7, and 14 days after TBI. For the bulk RNA-seq time-course analysis, n = 3 mice per time point.

To further define the temporal dynamics of cGAS–STING pathway alterations after injury, transcriptomic data from the TBI model were analyzed across different post-injury time points. Expression profiling revealed progressive activation in cGAS–STING pathway-related genes, with peak activation occurring at 3 days after injury (Fig. 2H). Consistently, gene set enrichment analysis (GSEA) demonstrated activation of multiple immune-associated pathways following TBI, including Toll-like receptor signaling, NOD-like receptor signaling, and cytosolic DNA-sensing pathways involving cGAS–STING signaling (Fig. 2E, I, and J).

### Molecular docking and CETSA evaluate potential interactions between CGA and cGAS–STING pathway proteins

To explore whether CGA might interact with different levels of the core cGAS–STING signaling cascade, molecular docking was performed using mouse-derived structures of cGAS, STING, TBK1, and IRF3, which represent the principal sequential components of this pathway. Among the four proteins, STING showed the most favorable predicted binding energy (−8.76 kcal/mol), followed by cGAS (−8.372 kcal/mol), TBK1 (−8.178 kcal/mol), and IRF3 (−6.651 kcal/mol) (Fig. 3A–D). These values suggested comparatively favorable predicted interactions with the upstream components STING and cGAS, although docking energies alone cannot establish binding preference or direct target engagement. We therefore further evaluated these candidate proteins using CETSA. Two-way repeated-measures ANOVA revealed significant treatment-by-temperature interactions for cGAS (*P* = 0.0012) and STING (*P* = 0.0151). Sidak's multiple comparisons test further showed significant differences between CGA- and DMSO-treated samples at 46 °C for cGAS (adjusted *P* = 0.0485) and at 49 °C for STING (adjusted *P* = 0.0444). No significant overall thermal shift was observed for TBK1 or IRF3 under the tested conditions (Fig. 3E–I). Together, these results prioritize cGAS and STING as candidate proteins associated with CGA action, while not excluding indirect or multi-target regulation of the cGAS–STING signaling cascade.

**Fig. 3:**
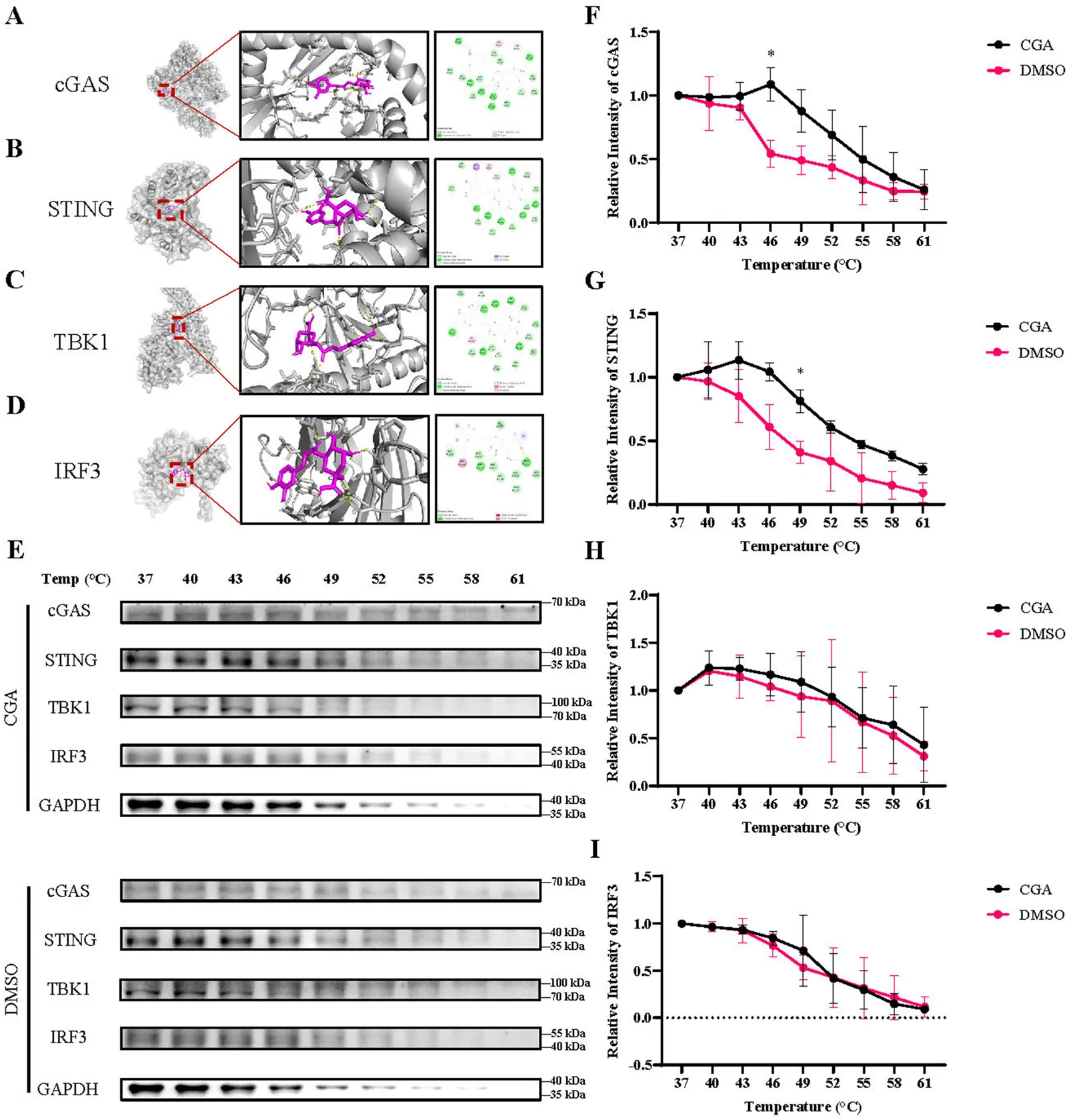
Molecular docking and CETSA evaluate potential interactions between CGA and components of the cGAS–STING pathway. (A–D) Predicted binding conformations and ligand–protein interactions between CGA and murine cGAS (A), STING (B), TBK1 (C), and IRF3 (D). (E) Representative CETSA immunoblots of cGAS, STING, TBK1, and IRF3 in CGA- or DMSO-treated samples across the indicated temperature gradient. (F–I) Quantification of the thermal stability profiles of cGAS (F), STING (G), TBK1 (H), and IRF3 (I). Data are presented as mean ± SD from n = 3 independent experiments. Thermal stability curves were analyzed using two-way repeated-measures ANOVA with Geisser–Greenhouse correction, followed by Sidak's multiple comparisons test. Asterisks above individual temperatures indicate significant Sidak-adjusted comparisons between CGA- and DMSO-treated samples at the corresponding temperature. *P* < 0.05.

### Single-cell transcriptomic analysis reveals cell-type-specific cGAS–STING activity after TBI

To characterize the cellular distribution of cGAS–STING signaling after TBI, we analyzed a publicly available murine single-cell RNA-seq dataset containing sham and TBI samples collected at 1, 3, 7, and 14 dpi. Following quality-control filtering and dimensionality reduction, cells were annotated into 11 major neural, immune, and vascular/stromal populations based on canonical marker genes (Supplementary Fig. 3A–D, F). Analysis of cellular composition revealed time-dependent changes in the relative proportions of these populations after TBI (Supplementary Fig. 3E). Cell-type-specific t-SNE visualization further demonstrated the distribution of the annotated populations across individual time points, and projection of microglia provided a direct visualization of their distribution after injury (Supplementary Fig. 3F and G). cGAS–STING activity scores were subsequently compared across cell populations and time points. Higher pathway scores were observed in microglia and macrophages relative to most other identified cell populations, with dynamic changes during the post-injury period (Fig. 2K and L).

To further examine the cellular localization of cGAS–STING pathway-associated signals in the injured brain, immunofluorescence co-localization analysis was performed in the pericontusional cortex. cGAS, p-STING, and *p*-IRF3 signals were observed in IBA-1-positive cells after TBI. Quantitative analysis showed increased mean fluorescence intensities of cGAS, p-STING, and *p*-IRF3 in IBA-1-positive cells after injury, whereas CGA treatment significantly reduced these signals (Supplementary Fig. 4A–E). Because IBA-1 does not distinguish resident microglia from infiltrating macrophages, we additionally examined TMEM119 immunoreactivity after TBI. TMEM119-positive cells were readily detected in sham and contralateral brain regions, whereas TMEM119 immunoreactivity was visibly reduced in the ipsilateral injured cortex (Supplementary Fig. 5A). This pattern is consistent with previous observations that TMEM119 protein expression decreases in activated microglia near the contusion after TBI [36]. Co-immunofluorescence further showed that cGAS, p-STING, and *p*-IRF3 signals were present in a subset of TMEM119-positive cells within the ipsilateral hemisphere (Supplementary Fig. 5B). However, given the injury-associated reduction in TMEM119 expression, these findings were not used to exclude a contribution from infiltrating macrophages.

### CGA inhibits TBI-induced cGAS–STING activation *in vivo*

The effects of CGA on cGAS–STING signaling after TBI were initially evaluated by transcriptomic analysis of pericontusional cortical tissue. Compared with vehicle-treated TBI mice, CGA treatment broadly reduced the expression of genes associated with the cGAS–STING pathway (Fig. 4A and B). To determine whether systemically administered CGA reached the injured brain, CGA concentrations were further measured in paired ipsilateral and contralateral brain tissues by LC–MS/MS. CGA was detectable in both hemispheres after intravenous administration, with significantly higher concentrations in the ipsilateral injured hemisphere than in the contralateral hemisphere (Fig. 4C). These findings demonstrate brain exposure to CGA after systemic administration in TBI mice. Consistent with these observations, Western blot analysis showed enhanced activation of the cGAS–STING pathway after TBI. Compared with sham controls, injured cortical tissue exhibited increased cGAS expression together with enhanced phosphorylation of STING, TBK1, and IRF3. CGA treatment reduced cGAS expression and decreased the phosphorylation levels of STING, TBK1, and IRF3 (Fig. 4D–F, H, I). The downstream inflammatory responses were evaluated by measuring IL-1β and IL-6 levels in cortical homogenates. Both cytokines were increased after TBI and were significantly reduced by CGA administration (Fig. 4G and J).

**Fig. 4:**
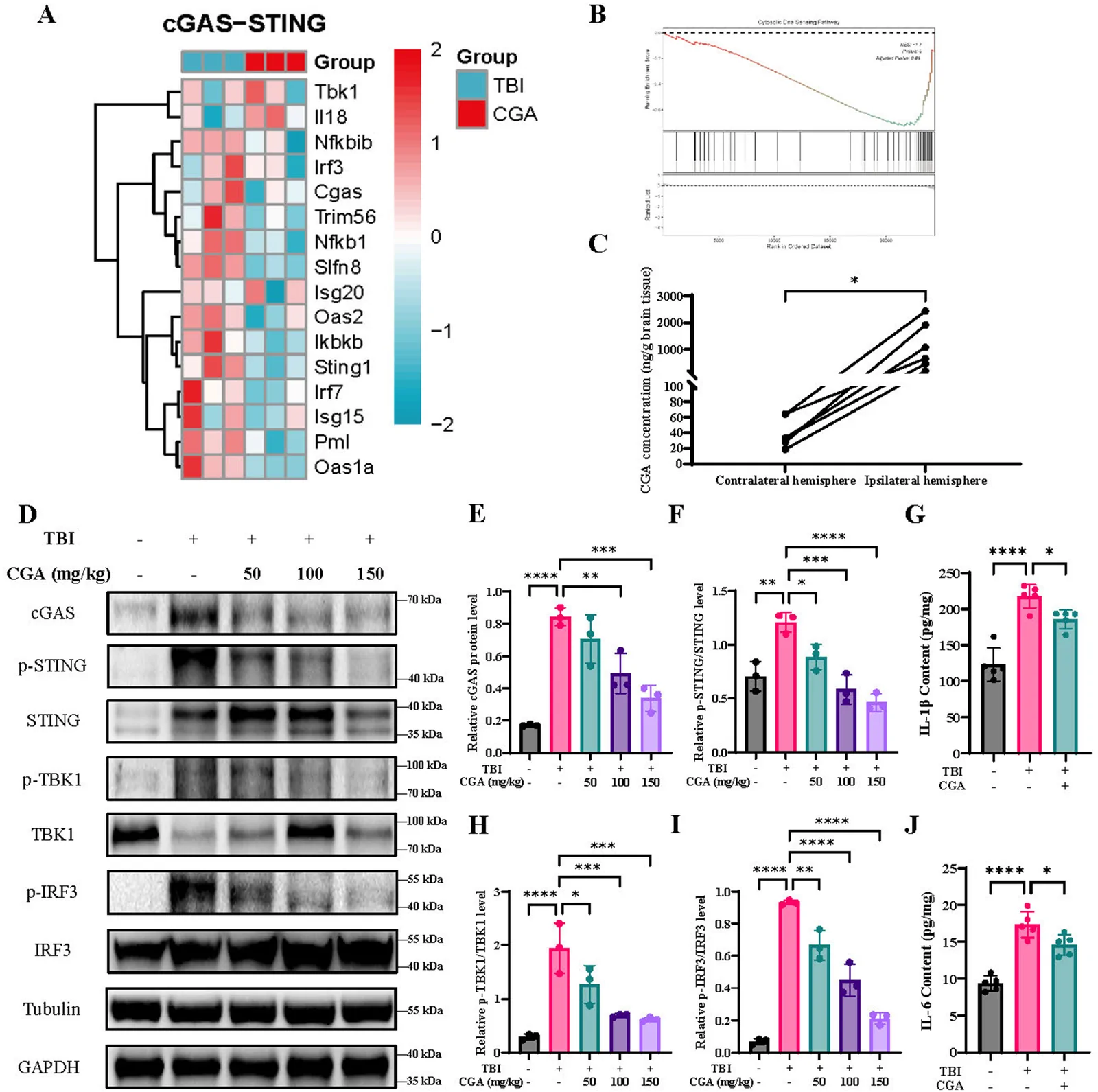
Systemic CGA administration suppresses TBI-induced cGAS–STING activation and downstream inflammation *in vivo*. (A) Hierarchical clustering heatmap of cGAS–STING-related genes in cortical tissue from vehicle- and CGA-treated TBI mice. (B) GSEA of the cytosolic DNA-sensing pathway following CGA treatment. (C) LC–MS/MS quantification of CGA concentrations in paired contralateral and ipsilateral brain tissue following systemic CGA administration. (D) Representative Western blots showing cGAS expression and phosphorylation of STING, TBK1, and IRF3 in sham, vehicle-treated TBI, and CGA-treated TBI mice. (E, F, H, I) Densitometric quantification of cGAS, p-STING/STING, *p*-TBK1/TBK1, and *p*-IRF3/IRF3, respectively. (G, J) ELISA quantification of IL-1β and IL-6 in brain tissue. Data are presented as mean ± SD. For western blotting, n = 3 mice per group; for ELISA, n = 5 mice per group; for LC–MS/MS, n = 6 mice with paired ipsilateral and contralateral samples. The paired comparison of CGA concentrations between ipsilateral and contralateral hemispheres (C) was performed using a two-tailed paired *t*-test. Other multi-group comparisons were performed using one-way ANOVA followed by Dunnett's multiple comparisons test. ∗*P* < 0.05, ∗∗*P* < 0.01, ∗∗∗*P* < 0.001, and ∗∗∗∗*P* < 0.0001.

### CGA reduces inflammatory responses and oxidative stress in LPS-stimulated microglia

The anti-inflammatory effects of CGA were further evaluated in LPS-stimulated BV2 microglia. CGA showed no detectable cytotoxicity within the concentrations used in this study (Fig. 5A). LPS markedly increased the expression of pro-inflammatory mediators in BV2 cells. CGA treatment reduced the mRNA expression of *Il1b*, *Il6*, and *Tnf* in a concentration-dependent manner (Fig. 5B, C, E). In addition, CGA decreased the expression of inflammation-associated enzymes, including *Cox2* and *Nos2* (Fig. 5F and G). Intracellular ROS accumulation was then assessed using the DCFH-DA fluorescent probe. Flow cytometry demonstrated that LPS markedly increased the proportion of ROS-positive cells, whereas CGA treatment significantly reduced ROS accumulation (Fig. 5D, H, and I).

**Fig. 5:**
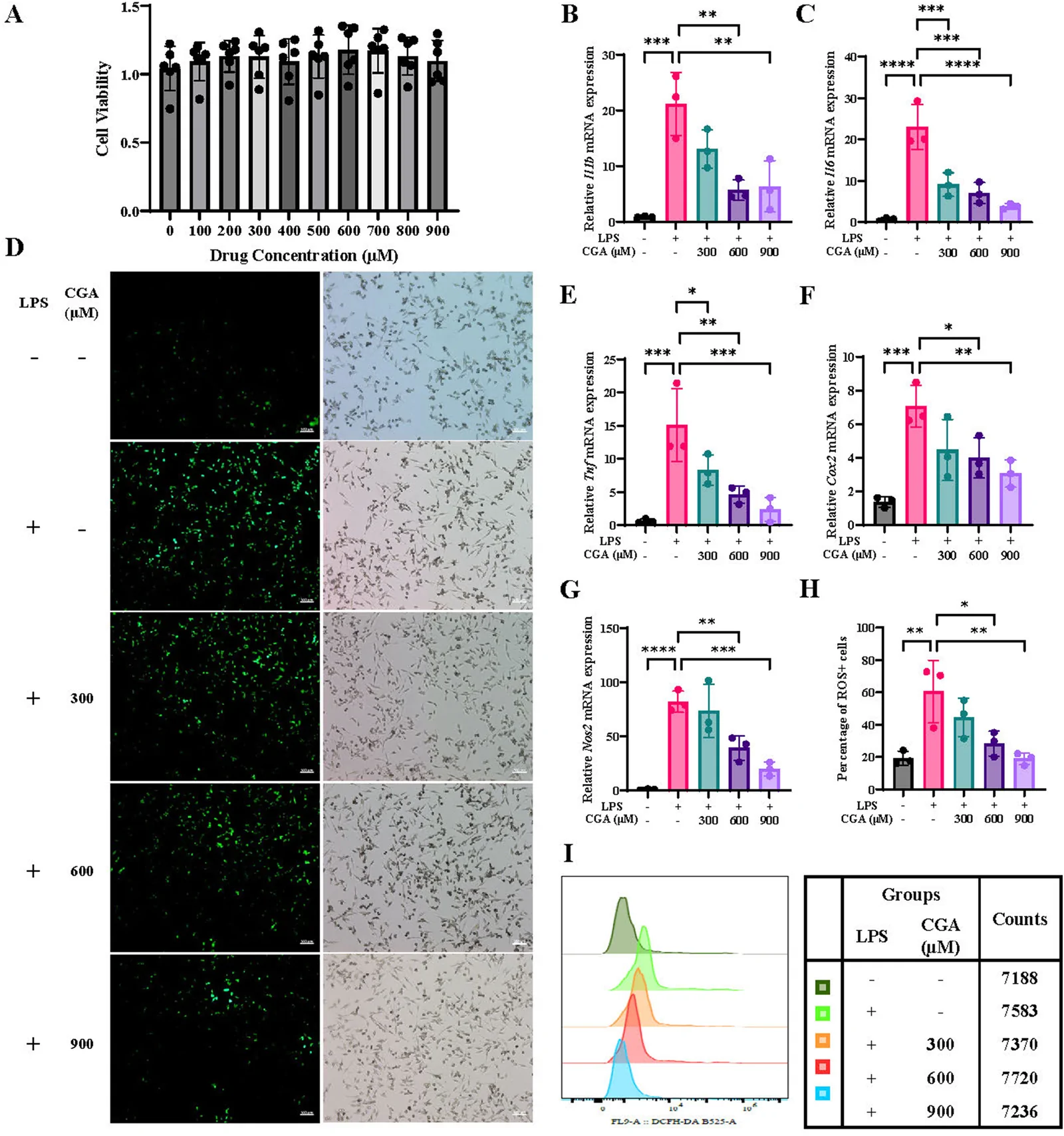
CGA attenuates LPS-triggered pro-inflammatory responses and ROS accumulation in BV2 microglia *in vitro*. (A) CCK-8 cell viability profile of BV2 cells exposed to different concentrations of CGA (0–900 μM). (B, C, E, F, G) Quantitative real-time PCR (qRT-PCR) determination of relative mRNA expression levels for *Il1b*, *Il6*, *Tnf*, *Cox2*, and *Nos2*. (D) Representative immunofluorescence and brightfield microscopic images of intracellular ROS accumulation in BV2 cells. (H) Quantitative evaluation of ROS-positive cells. (I) Representative flow cytometry histograms. Data are presented as mean ± SD from n = 3 independent biological replicates. Statistical comparisons were performed using one-way ANOVA followed by Dunnett's multiple comparisons test. ∗*P* < 0.05, ∗∗*P* < 0.01, ∗∗∗*P* < 0.001, and ∗∗∗∗*P* < 0.0001.

### CGA attenuates cGAS–STING-associated signaling in LPS-stimulated microglia

To examine whether CGA modulates cGAS–STING-associated responses under inflammatory conditions, pathway-related molecular changes were evaluated in LPS-stimulated BV2 cells. Transcriptomic profiling showed that CGA broadly reduced the expression of genes involved in STING-mediated inflammatory signaling (Fig. 6A and D). Downstream transcriptional responses to cGAS–STING activation were further assessed by quantitative real-time PCR. LPS stimulation markedly increased the expression of interferon-stimulated genes (ISGs), including *Isg15*, *Isg20*, *Ifit1*, *Ifnb1*, and *Cxcl10*. CGA treatment significantly reduced the expression levels of these ISGs in a concentration-dependent manner (Fig. 6B, C, E, G, H).

**Fig. 6:**
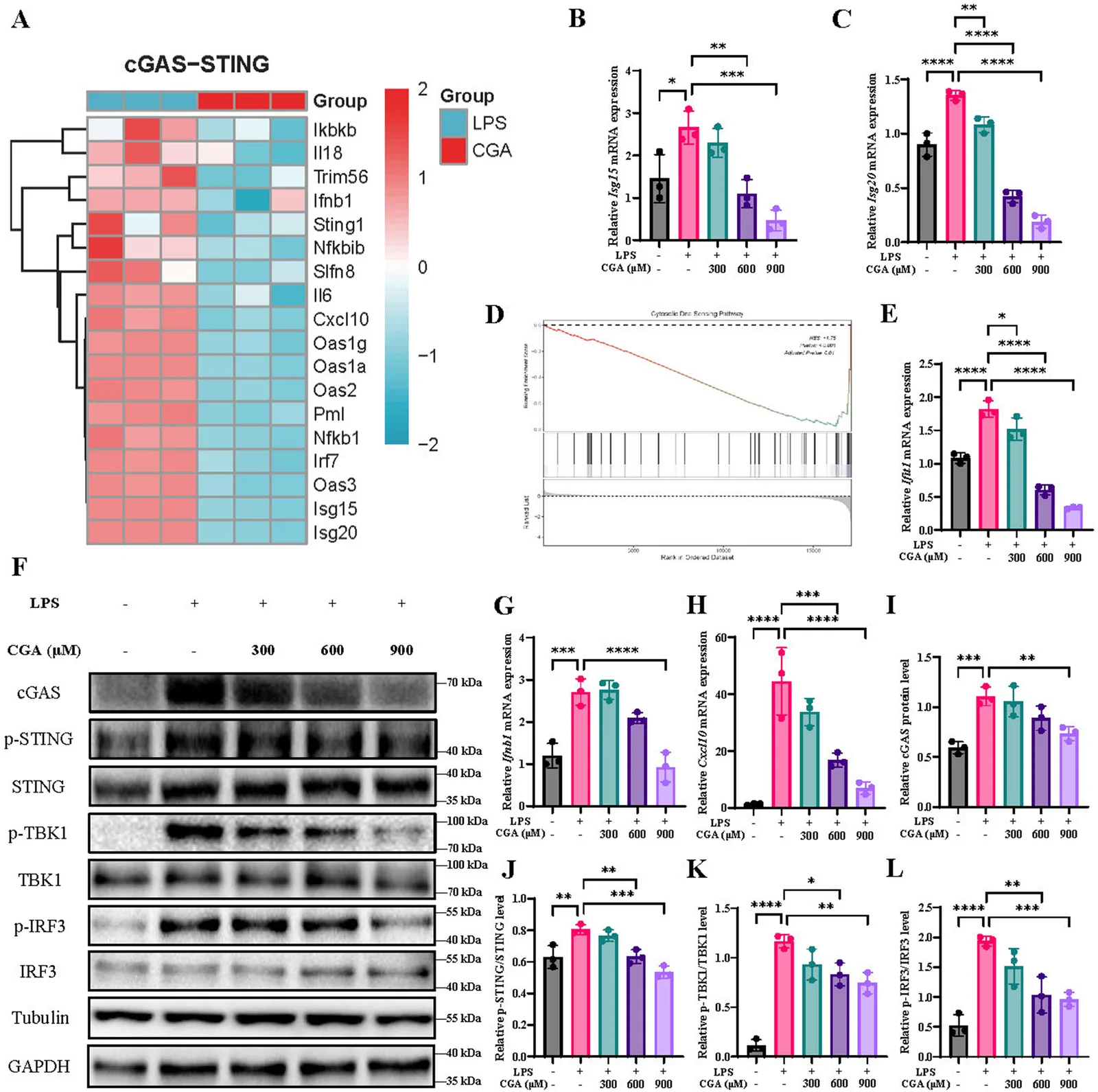
CGA inhibits cGAS–STING signaling and suppresses downstream interferon signaling. (A) Heatmap showing changes in cGAS–STING-related gene expression after CGA treatment. (B, C, E, G, H) qRT-PCR quantification of the indicated interferon-stimulated genes. (D) GSEA of cGAS–STING-associated signaling following CGA treatment. (F) Representative immunoblots showing cGAS expression and phosphorylation of STING, TBK1, and IRF3. (I–L) Densitometric quantification of cGAS, p-STING/STING, *p*-TBK1/TBK1, and *p*-IRF3/IRF3, respectively. Data are presented as mean ± SD from n = 3 independent biological replicates. Statistical comparisons were performed using one-way ANOVA followed by Dunnett's multiple comparisons test. ∗*P* < 0.05, ∗∗*P* < 0.01, ∗∗∗*P* < 0.001, and ∗∗∗∗*P* < 0.0001.

Consistent with these changes at the transcriptional level, Western blot analysis revealed that LPS stimulation was accompanied by increased cGAS expression and enhanced phosphorylation of STING, TBK1, and IRF3. CGA treatment reduced cGAS expression and the phosphorylation levels of these downstream pathway components (Fig. 6F, I–L).

### STING activation counteracts CGA-mediated suppression of microglial inflammation

To examine whether pharmacological activation of STING could counteract the inhibitory effects of CGA, rescue experiments were performed using DMXAA in LPS-stimulated BV2 microglia. LPS stimulation increased the expression of pro-inflammatory and interferon-responsive genes, whereas CGA treatment reduced *Il1b*, *Il6*, *Nos2*, *Tnf*, *Cxcl10*, *Isg15*, *Ifit1*, and *Ifnb1* expression (Fig. 7A–H). Addition of DMXAA to CGA-treated cells partially reversed these transcriptional effects. Consistent with the qRT-PCR results, Western blot analysis showed that DMXAA restored STING, TBK1, and IRF3 phosphorylation in the presence of CGA (Fig. 7I–M). Notably, the p-STING/STING, *p*-TBK1/TBK1, and *p*-IRF3/IRF3 ratios did not differ significantly between the LPS + CGA + DMXAA and LPS + DMXAA groups (Fig. 7K–M), indicating that CGA did not significantly suppress downstream pathway activation once STING was pharmacologically activated by DMXAA. These findings are consistent with CGA acting at or upstream of STING, rather than directly inhibiting downstream TBK1 or IRF3 activation, although they do not establish an exclusive site of action.

**Fig. 7:**
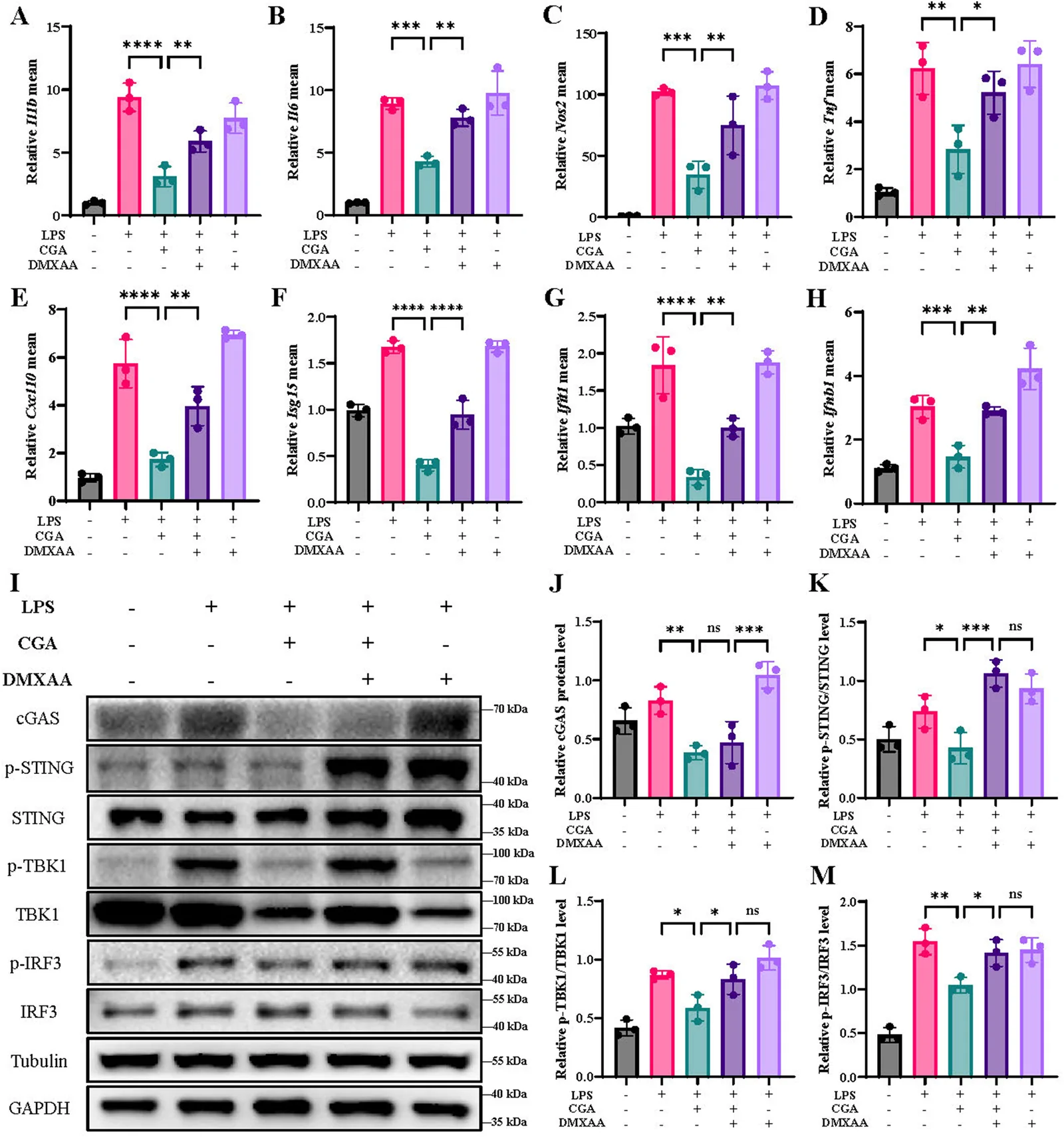
Pharmacological reactivation of STING reverses the anti-inflammatory effects of CGA. (A–H) qRT-PCR quantification showing that DMXAA partially reversed CGA-mediated suppression of inflammatory and interferon-stimulated gene expression. (I) Representative immunoblots showing restoration of STING, TBK1, and IRF3 phosphorylation by DMXAA in the presence of CGA. (J–M) Densitometric quantification of cGAS expression and the p-STING/STING, *p*-TBK1/TBK1, and *p*-IRF3/IRF3 ratios. Data are presented as mean ± SD from n = 3 independent biological replicates. Statistical comparisons were performed using one-way ANOVA followed by Sidak's multiple comparisons test. ns, not significant; ∗*P* < 0.05, ∗∗*P* < 0.01, ∗∗∗*P* < 0.001, and ∗∗∗∗*P* < 0.0001.

## Discussion

Traumatic brain injury initiates a complex secondary injury cascade in which a persistent neuroinflammatory response plays a significant role in progressive tissue damage and neurological dysfunction. Among the cellular components involved in this process, microglia serve as key regulators of the inflammatory environment within the injured central nervous system. Excessive or prolonged microglial activation can amplify inflammatory signaling and oxidative stress, thereby extending the consequences of the initial mechanical insult. Related studies in other forms of traumatic CNS injury have further demonstrated that oxidative stress, mitochondrial dysfunction, inflammasome activation, autophagy dysregulation, endoplasmic reticulum stress, and pyroptosis can interact to shape secondary injury and neurological recovery [[37], [38], [39]]. In the present study, complementary transcriptomic, computational, and experimental approaches were used to investigate the mechanisms underlying the effects of CGA after TBI. Collectively, the findings support suppression of microglial cGAS–STING signaling as a mechanism that may contribute to the anti-inflammatory effects of CGA [11,18,40].

The cGAS–STING pathway has emerged as an important DNA-sensing mechanism linking cellular damage to innate immune activation and has increasingly been implicated in neuroinflammation and regenerative responses after nervous system injury [41]. Under physiological conditions, cytosolic DNA is tightly controlled. However, mitochondrial dysfunction, nuclear damage, and cellular stress after tissue injury can lead to the accumulation of endogenous DNA fragments, thereby triggering cGAS activation and subsequent STING-dependent signaling [12,42,43]. Activation of this pathway induces downstream TBK1 activation and IRF3 phosphorylation, resulting in the production of type I interferons and inflammatory mediators that participate in secondary injury processes. Consistent with previous observations in CNS injury models, our transcriptomic and experimental analyses revealed activation of the cGAS–STING pathway following TBI [15]. Single-cell transcriptomic analysis revealed elevated cGAS–STING activity in several immune cell populations after TBI, including microglia and infiltrating macrophages. These findings indicate that resident microglia are not the exclusive cellular source of pathway activation. Our subsequent mechanistic experiments focused on microglia because they are resident CNS immune cells with sustained involvement in post-traumatic neuroinflammation and because the *in vitro* experiments were designed using a microglial model. The distinction between resident microglia and infiltrating macrophages is further complicated by injury-associated changes in commonly used microglial markers. In our tissue sections, TMEM119 immunoreactivity was reduced in the ipsilateral injured cortex compared with sham and contralateral regions. This observation is consistent with the study by Mercurio et al. who showed that TMEM119 protein expression and immunoreactivity decrease in activated Iba1-positive myeloid cells near the contusion after experimental TBI, particularly in hypertrophic and amoeboid cells [36]. Consequently, TMEM119 staining alone cannot reliably exclude infiltrating macrophages in the injured cortex. We therefore interpret the immunofluorescence findings as supporting the involvement of microglia, while acknowledging that macrophages and other infiltrating immune cells may also contribute to cGAS–STING activation after TBI. Nevertheless, the contribution of infiltrating macrophages and other immune populations remains to be determined. These findings support the contribution of microglial innate immune signaling to the regulation of post-traumatic inflammatory responses and suggest that microglial cGAS–STING activity may influence the transition between resolving and persistent neuroinflammation [[44], [45], [46]].

The behavioral findings suggest that the protective effects of CGA extend beyond acute neurological recovery and may influence later functional outcomes after TBI. Importantly, the absence of significant differences in spontaneous locomotor activity among groups reduces, although does not eliminate, the possibility that the improved Morris water maze performance simply reflected altered motor capacity. Given the established contribution of neuroinflammation to secondary neurological dysfunction after TBI, early suppression of inflammatory signaling may contribute to the later functional benefits observed after CGA treatment [47]. However, because cGAS–STING activity was assessed primarily during the acute phase, the present data do not establish a direct causal relationship between early pathway inhibition and long-term cognitive improvement.

One strength of the present study is the integrated strategy used to identify the molecular pathway associated with the effects of CGA after TBI. Instead of relying solely on direct overlap between predicted drug targets and disease-related genes, multiple analytical approaches were combined to prioritize candidate pathways involved in the response to CGA. Sequential filtering using different topological algorithms identified a group of highly connected hub genes associated with inflammatory signaling. Subsequent enrichment analyses linked these candidates to innate immune pathways, particularly the cGAS–STING signaling axis. Molecular docking was used to examine potential interactions between CGA and several components of the cGAS–STING cascade. The predicted binding energies showed a hierarchical pattern, with STING exhibiting the most favorable value, followed by cGAS, TBK1, and IRF3. Consistent with this computational prioritization, CETSA revealed changes in the thermal stability of cGAS and STING after CGA exposure, whereas comparable shifts were not detected for TBK1 or IRF3 under the tested conditions. These findings suggest that the upstream cGAS–STING components may represent more plausible candidates for CGA-associated target engagement. Nevertheless, neither molecular docking nor CETSA alone is sufficient to establish the primary direct molecular target of CGA. The present findings therefore remain compatible with a multi-target or pathway-level mechanism, and further orthogonal biophysical validation will be required to distinguish direct binding from indirect stabilization and to define the relative contribution of individual targets.

Pharmacological rescue experiments further provided functional evidence linking cGAS–STING signaling to the anti-inflammatory effects of CGA. In LPS-stimulated BV2 microglia, CGA reduced the production of inflammatory mediators, interferon-stimulated genes, and intracellular ROS accumulation. Pharmacological activation of STING by DMXAA [48] partially reversed these inhibitory effects and restored downstream STING/TBK1/IRF3 signaling activity. However, DMXAA was evaluated at only one concentration in the present rescue experiments. Therefore, these findings should be interpreted as preliminary pharmacological evidence consistent with the involvement of STING signaling, rather than definitive evidence of concentration-dependent or exclusive STING dependence. Future studies using graded concentrations of DMXAA and genetic loss-of-function approaches targeting cGAS or STING will be required to strengthen causal inference. These observations indicate that inhibition of the cGAS–STING pathway is involved in the cellular response to CGA and suggest that modulating innate immune signaling contributes to its anti-inflammatory properties.

CGA also reduced intracellular ROS accumulation in LPS-stimulated BV2 cells. Oxidative stress and cGAS–STING signaling may be mechanistically interconnected, as mitochondrial stress can promote the release of mitochondrial DNA into the cytosol and thereby provide endogenous DNA ligands for cGAS activation [42,43]. However, the present experiments did not directly examine whether the reduction in ROS occurred upstream or downstream of cGAS–STING inhibition, or whether the two changes represented parallel effects of CGA. Accordingly, the reduction in ROS should be interpreted as an associated cellular effect rather than evidence for a defined upstream–downstream relationship. Further experiments specifically manipulating oxidative stress or cytosolic DNA signaling will be required to resolve this relationship.

From a translational perspective, several issues require consideration before CGA can be evaluated as a therapeutic strategy for TBI. In the present study, CGA was administered intravenously during the early post-injury period. Although this route provides controlled systemic exposure in experimental animals, its feasibility may be limited in some patients with acute TBI because of heterogeneous injury severity and systemic instability. Previous pharmacokinetic studies have also demonstrated detectable CGA in the CSF and brain following intravenous administration, although CNS penetration appears limited under non-injured conditions. For example, a rat UHPLC–MS/MS study reported a CSF-to-plasma exposure ratio of approximately 0.29% after intravenous CGA administration [49]. Importantly, our additional LC–MS/MS analysis detected CGA in brain tissue after intravenous administration, with higher concentrations in the ipsilateral injured hemisphere than in the contralateral hemisphere, indicating that systemically administered CGA can reach the injured brain. However, these data should not be interpreted as evidence that CGA efficiently crosses an intact blood–brain barrier, particularly because BBB permeability is altered after TBI. For longer-term treatment, oral administration may be more practical, but its systemic bioavailability and resulting brain exposure require dedicated pharmacokinetic evaluation. Translation of the doses used in mice will also require appropriate human-equivalent dose estimation and subsequent pharmacokinetic and dose-escalation studies rather than direct conversion based on body weight alone. In addition, engineered delivery platforms, nano-formulations, or prodrug strategies may provide opportunities to improve central nervous system exposure and reduce systemic dosing requirements. Recent advances in spatiotemporally controlled drug-delivery platforms for nervous system injury illustrate the potential of such approaches for remodeling the local injury microenvironment [50]. Further studies defining the pharmacokinetics, optimal route of administration, therapeutic window, dosing schedule, and safety of CGA will therefore be necessary before clinical translation can be considered. Recent studies using biomaterial-based delivery platforms in CNS injury models, including conductive hydrogels and mesoporous polydopamine nanoparticles, further illustrate the potential of engineered delivery systems to modulate the post-injury microenvironment and improve therapeutic efficacy [51,52]. These pharmacokinetic and delivery studies, together with validation in clinically relevant TBI models, will be important steps toward translating the present findings into therapeutic development.

Several limitations should be considered when interpreting the present findings. First, molecular analyses of cGAS–STING signaling were performed primarily during the acute phase after TBI, whereas spatial learning and memory were evaluated at later time points. Therefore, although early pathway suppression was associated with improved long-term behavioral outcomes, the present study does not establish a direct temporal or causal link between these observations. Second, although the present study focused on microglia, single-cell analysis also revealed substantial cGAS–STING activity in infiltrating immune populations, particularly macrophages. Therefore, the current data do not establish microglia as the exclusive or predominant cellular source of pathway activation after TBI. Future studies using microglia-specific conditional deletion of *Cgas* or *Sting1*, for example by crossing inducible microglial Cre-driver lines with *Cgas-* or *Sting1-*floxed mice, will be required to determine whether microglial cGAS–STING signaling is necessary for the protective effects of CGA. Third, the *in vitro* experiments were performed using LPS-stimulated BV2 microglia, which provide a reproducible reductionist model of inflammatory activation but do not fully reproduce the molecular phenotype of primary microglia or the complex pathological microenvironment of TBI. Validation in primary microglia will therefore be important in future mechanistic studies. In particular, LPS primarily initiates inflammatory signaling through TLR4, whereas cellular and mitochondrial damage after TBI can generate cytosolic DNA that directly engages the cGAS–STING pathway. Previous studies have shown that LPS can also induce mitochondrial dysfunction, oxidative stress, and cytosolic mitochondrial DNA release in BV2 microglia, providing a plausible indirect link between LPS-induced inflammatory stress and cGAS–STING activation. However, mitochondrial DNA release was not directly examined in the present experiments; therefore, the BV2/LPS model was used to assess the ability of CGA to modulate inflammatory and cGAS–STING-associated responses rather than to recapitulate the complete upstream mechanism of cGAS activation after TBI [53]. Future studies using primary microglia and injury-relevant stimuli, including cytosolic DNA or other damage-associated signals, will be valuable for further validation. In addition, the pharmacological rescue experiments were performed using a single concentration of DMXAA. Although the partial reversal of CGA-mediated effects supports the involvement of STING signaling, concentration-dependent pharmacological studies and genetic manipulation of cGAS or STING will be required to strengthen causal inference. Furthermore, although molecular docking predicted favorable interactions between CGA and several components of the cGAS–STING pathway, computational predictions cannot establish direct target engagement. Although the CETSA results provide additional evidence of CGA-associated target engagement with cGAS and STING, this assay does not by itself establish direct physical binding or define a single primary molecular target. Orthogonal biophysical approaches, such as surface plasmon resonance (SPR) or microscale thermophoresis (MST), together with structural studies where appropriate, will be required to further characterize the molecular basis of these interactions [54,55]. Finally, whether prolonged CGA administration can provide sustained neurological benefits and influence long-term pathological changes after injury remains to be determined.

Overall, our findings identify microglial cGAS–STING signaling as a potential mediator of post-traumatic neuroinflammation and provide evidence that CGA alleviates inflammatory responses and neurological deficits after TBI. The early suppression of cGAS–STING signaling observed after CGA treatment may contribute to subsequent functional recovery; however, the present data do not establish that this early molecular effect directly mediates long-term cognitive improvement. By integrating transcriptomic, single-cell, computational, and experimental approaches, we identified cGAS–STING signaling as a pathway associated with the anti-inflammatory effects of CGA after TBI. These findings support further investigation of CGA as a potential therapeutic candidate for targeting neuroinflammatory responses after TBI.

## Institutional review board statement

The animal study was approved by Institutional Animal Welfare and Ethics Committee, China-Japan Friendship Hospital, Beijing, China (Approval ID: ZRDWLL240035). The study was conducted in accordance with the local legislation and institutional requirements.

## Data availability statement

The authors declare that all shared data supporting the findings of this study are available within the article and its supplementary information files. The publicly available murine TBI single-cell RNA sequencing dataset analyzed in this study was obtained from the Gene Expression Omnibus (GEO) database under accession number GSE302863. The bulk RNA sequencing datasets (including the time-course and treatment profiles) are available from the corresponding author upon reasonable request.

## Credit author statement

Xu Yang: Conceptualization, Methodology, Writing - Original Draft, Visualization.

Kun He: Methodology, Formal analysis, Writing - Original Draft.

Yulian Zhang: Writing - Review & Editing, Methodology, Funding acquisition.

Hanhan Dang: Software, Visualization.

Zixi Wang: Formal analysis, Validation.

Yunsheng Zhang: Investigation, Validation.

Chuanpeng Zhang: Resources, Investigation.

Pengyu Chen: Data Curation, Software.

Tongbo Zhang: Visualization, Resources.

Li Zhang: Project administration.

Yanbing Yu: Funding acquisition, Writing - Review & Editing, Supervision.

## Declaration of generative AI and AI-assisted technologies in the manuscript preparation process

During the preparation of this work, the author(s) used Gemini to polish the language. After using this tool/service, the author(s) reviewed and edited the content as needed and assume full responsibility for the content of the published article.

## Funding

The authors declare that financial support was received for the research and/or publication of this article. This work was supported by National High Level Hospital Clinical Research Funding (2025-NHLHCRF-XKJS-A-01), National Natural Science Foundation of China (32300816, 32471035), Capital Health Development Research Special Project (2026-1-4061), the Elite Medical Professionals Project of China–Japan Friendship Hospital (ZRJY2024-QM07), Beijing Natural Science Foundation (L242112).

## Declaration of competing interest

The authors declare that they have no known competing financial interests or personal relationships that could have appeared to influence the work reported in this paper.

## Figures and Tables

**Supplementary Fig. 1:**
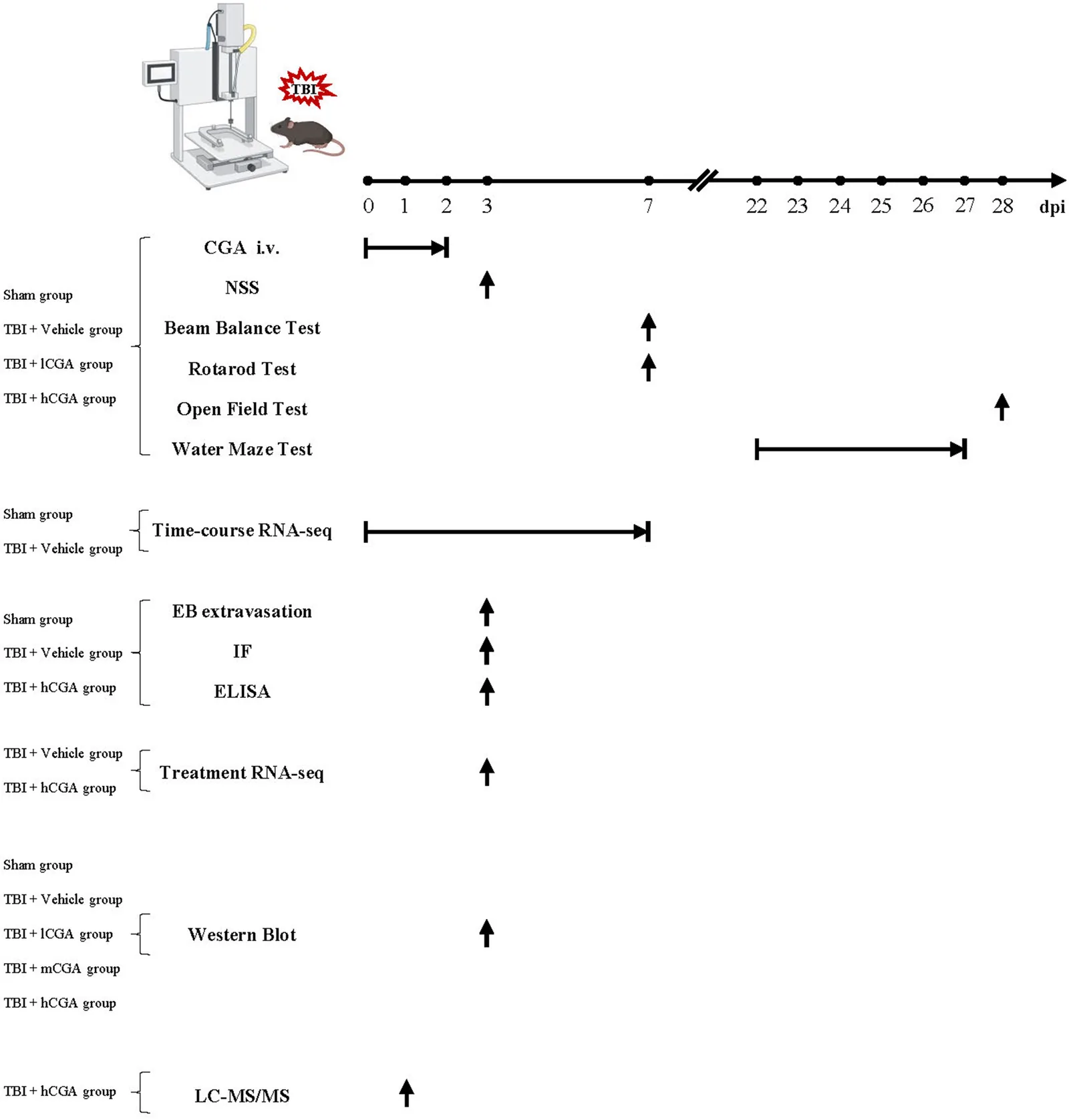
Experimental design and chronological workflow of the study. Schematic representation of the experimental groups. The timeline illustrates TBI induction by controlled cortical impact (CCI), sequential intravenous (i.v.) drug administration after injury, and the time points for subsequent assessments. These assessments include longitudinal neurobehavioral tests (NSS, Beam Balance, Rotarod, Open Field, and Morris Water Maze), bulk RNA sequencing, Evans Blue (EB) extravasation, western blotting, ELISA, and confocal immunofluorescence staining.

**Supplementary Fig. 2:**
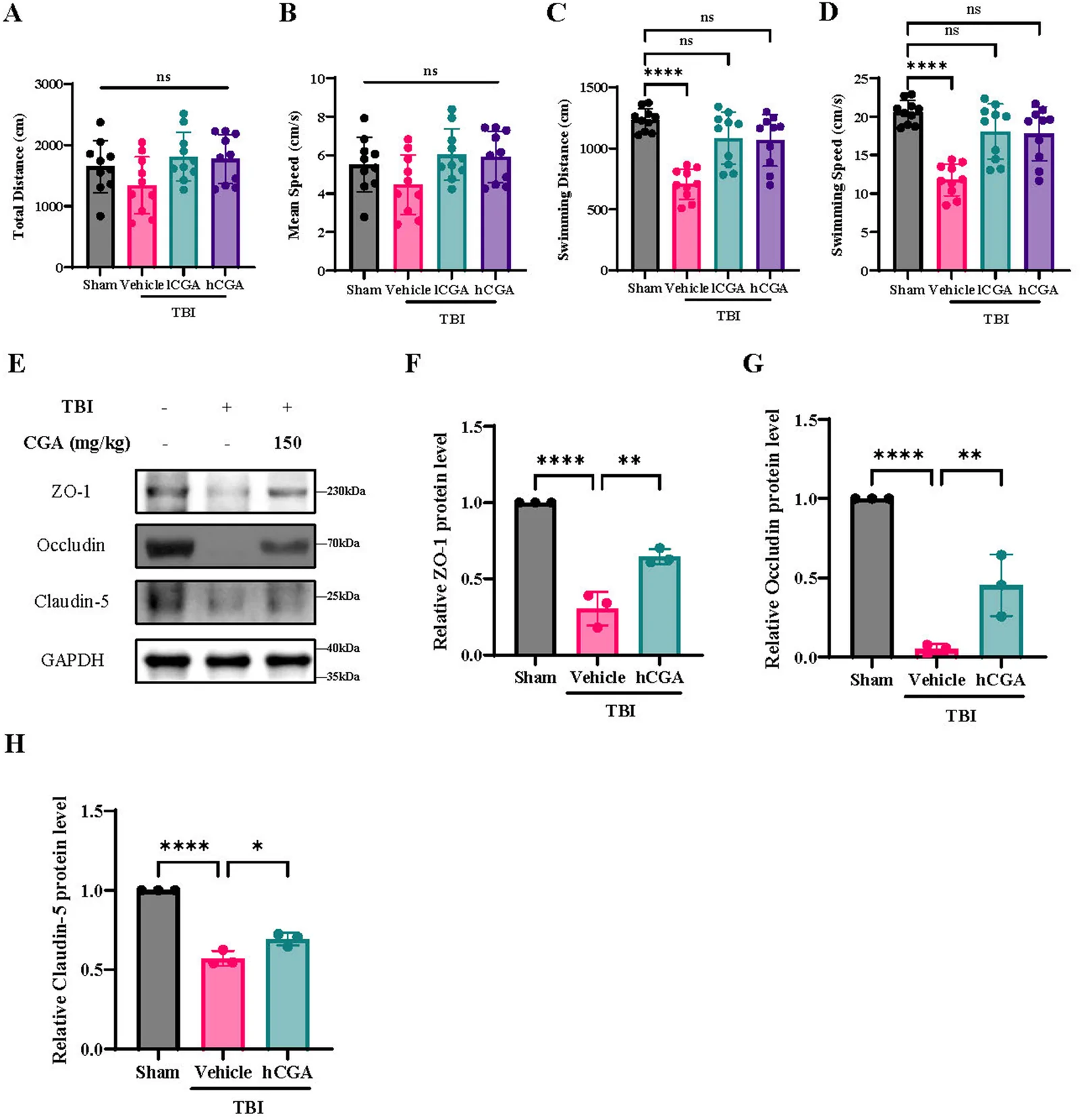
Supplementary behavioral assessments and tight-junction protein expression after TBI. Quantitative analysis of (A) total distance traveled and (B) mean speed during the open-field test. (C) Total swimming distance and (D) swimming speed during the Morris water maze probe trial. (E) Representative western blots showing ZO-1, Occludin, and Claudin-5 expression in sham, vehicle-treated TBI, and high-dose CGA-treated TBI mice. (F–H) Densitometric quantification of ZO-1 (F), Occludin (G), and Claudin-5 (H), normalized to GAPDH. Data are presented as mean ± SD. For behavioral assessments, n = 10 mice per group; for western blotting, n = 3 mice per group. ns, not significant; ∗*P* < 0.05, ∗∗*P* < 0.01, ∗∗∗*P* < 0.001, and ∗∗∗∗*P* < 0.0001.

**Supplementary Fig. 3:**
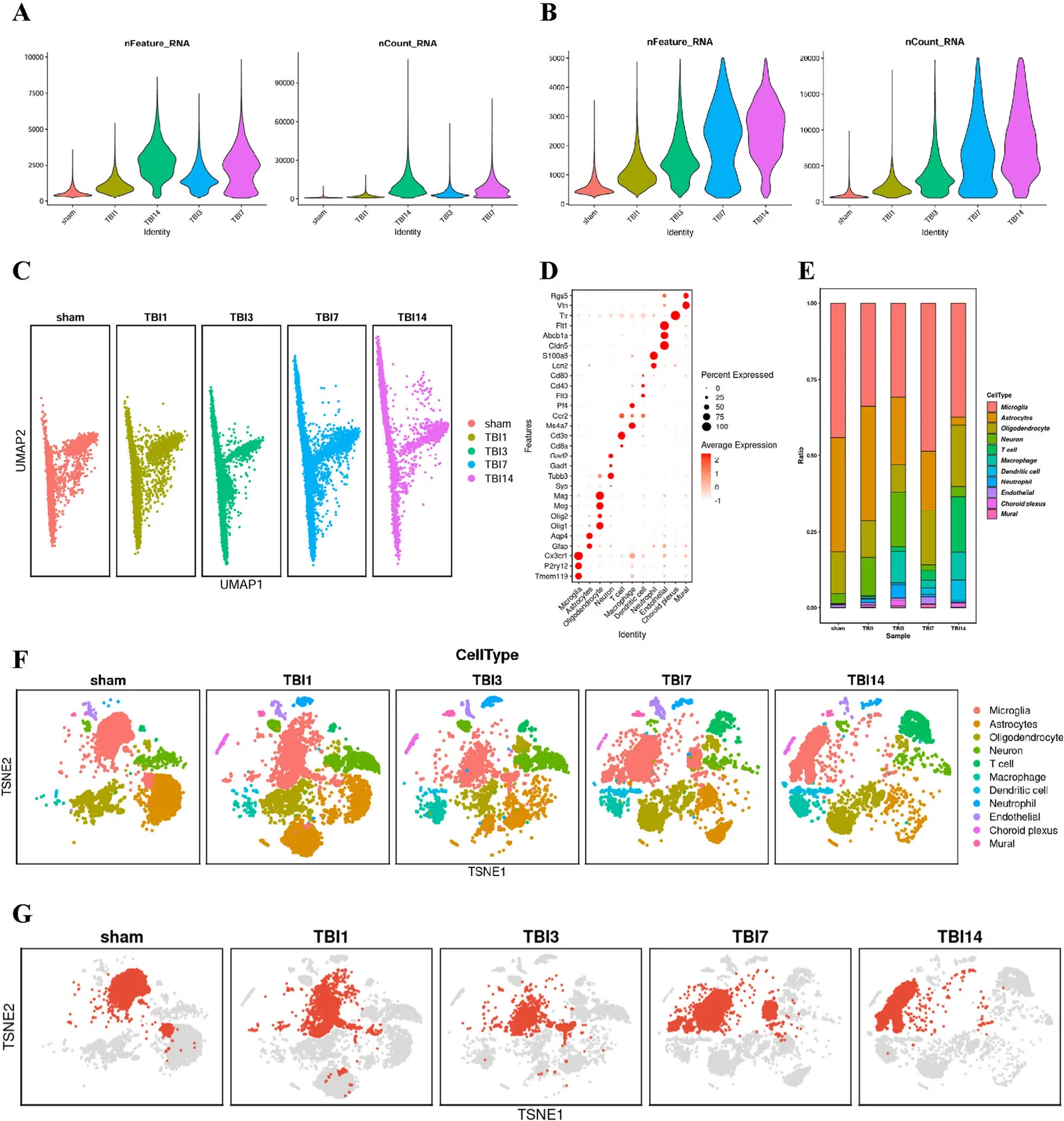
Quality control, cell-type annotation, and cellular composition of the single-cell RNA-seq dataset after TBI. (A, B) Violin plots showing the distributions of detected genes (nFeature_RNA) and total transcript counts (nCount_RNA) before and after quality-control filtering across sham and post-TBI samples. (C) UMAP visualization of single-cell transcriptomes from sham and TBI samples collected at 1, 3, 7, and 14 dpi. (D) Dot plot showing the expression of canonical marker genes used for cell-type annotation. (E) Relative proportions of the annotated cell populations across experimental time points. (F) t-SNE visualization of the major annotated CNS cell populations at each time point. (G) t-SNE projection highlighting the distribution of microglia across sham and post-TBI samples.

**Supplementary Fig. 4:**
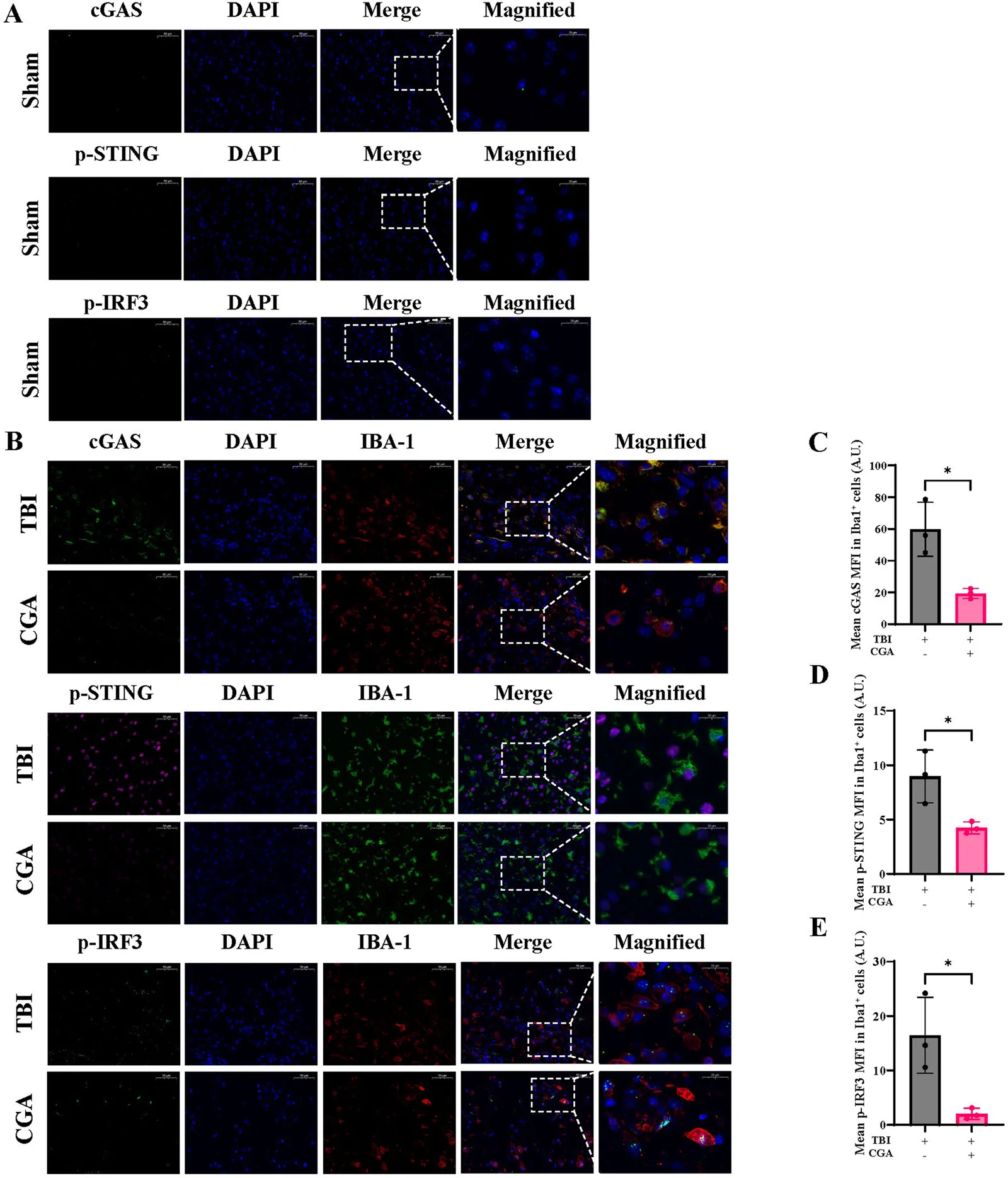
CGA reduces cGAS–STING pathway-associated immunofluorescence signals in IBA-1-positive cells after TBI. (A) Representative immunofluorescence images showing basal cGAS, p-STING, and *p*-IRF3 staining in sham brain tissue. (B) Representative co-immunofluorescence images of cGAS, p-STING, and *p*-IRF3 with IBA-1 in the pericontusional cortex of vehicle- and CGA-treated TBI mice. Enlarged regions are shown for visualization of cellular localization. (C–E) Quantification of mean fluorescence intensity (MFI) of cGAS (C), p-STING (D), and *p*-IRF3 (E) in IBA-1-positive cells. Data are presented as mean ± SD; n = 3 mice per group. ∗*P* < 0.05, ∗∗*P* < 0.01, ∗∗∗*P* < 0.001, and ∗∗∗∗*P* < 0.0001.

**Supplementary Fig. 5:**
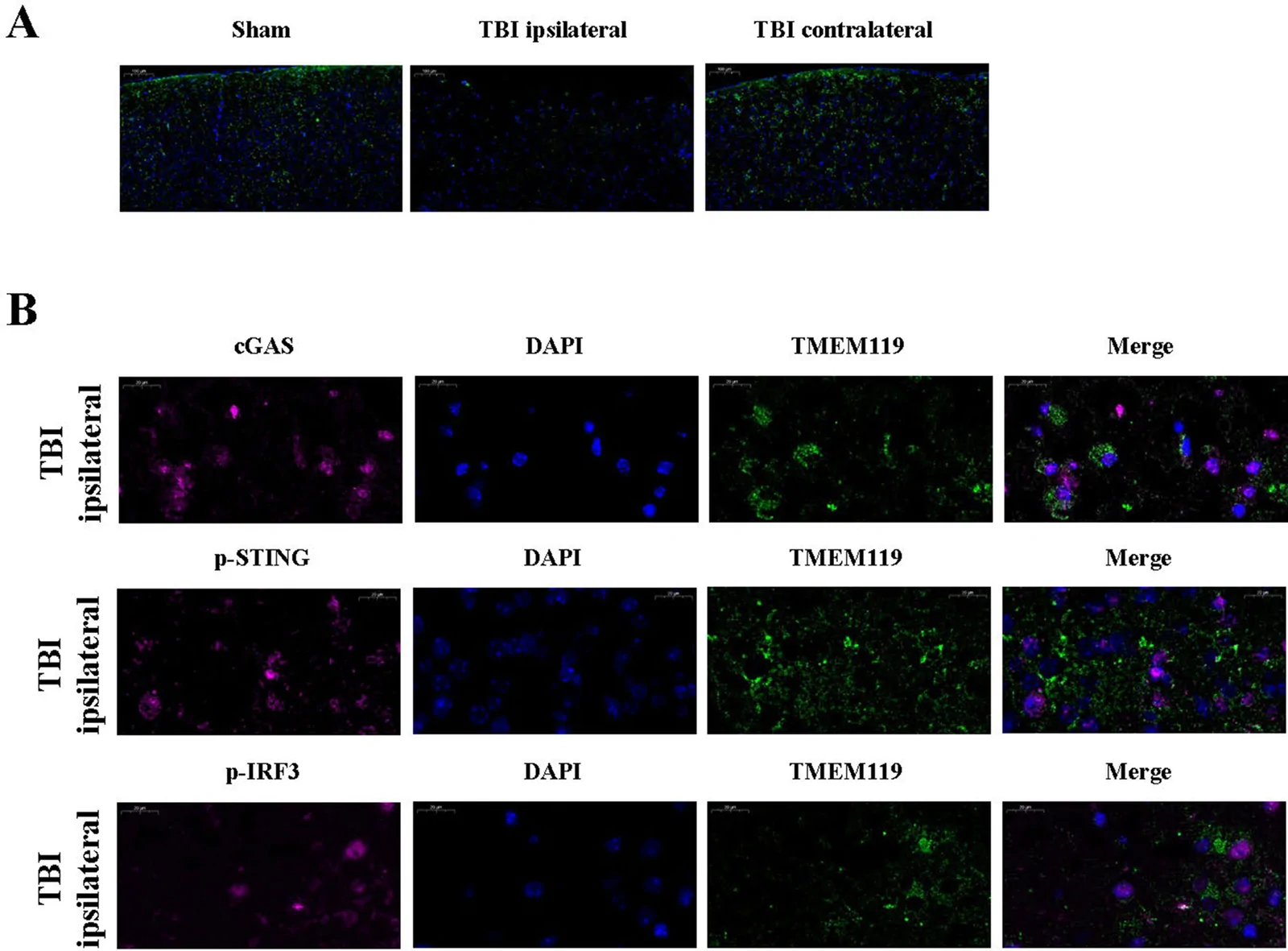
TMEM119 immunoreactivity and cGAS–STING pathway-associated signals after TBI. (A) Representative low-magnification images showing TMEM119 immunoreactivity in sham brain tissue and the contralateral and ipsilateral hemispheres after TBI. Reduced TMEM119 immunoreactivity was observed in the ipsilateral injured region. (B) Representative co-immunofluorescence images showing cGAS, p-STING, and *p*-IRF3 signals in TMEM119-positive cells within the ipsilateral hemisphere.
