# Supplementary material for: Chlorogenic acid attenuates TBI-induced neuroinflammation by suppressing cGAS–STING signaling
Source: Neurotherapeutics. 2026 Sep 28;23(6):e01085. doi: 10.1016/j.neurot.2026.e01085 (PMC13651564; doi:10.1016/j.neurot.2026.e01085)
Supplement: Supplementary file 1 [file mmc1.docx]

**Supplementary Table 1. Primer sequences used for quantitative real-time PCR.**

|  | Forward primer (5’-3’) | Reverse primer (5’-3’) |
| --- | --- | --- |
| *Gapdh* | AACTTTGGCATTGTGGAAGG | GGATGCAGGGATGATGTTCT |
| *Il1b* | TGTAATGAAAGACGGCACACC | TCTTCTTTGGGTATTGCTTGG |
| *Il6* | GCTACCAAACTGGATATAATCAGGA | CCAGGTAGCTATGGTACTCCAGAA |
| *Nos2* | GTTCTCAGCCCAACAATACAAGA | GTGGACGGGTCGATGTCAC |
| *Cox2* | GCTCAGCCAGGCAGCAAATC | ACCATAGAATCCAGTCCGGGT |
| *Tnf* | TGTAGCCCACGTCGTAGCAAA | CTTTGAGATCCATGCCGTTGG |
| *Cxcl10* | GTCTGAGTGGGACTCAAGGGAT | TCAACACGTGGGCAGGATAG |
| *Ifit1* | AAGGCTGTCCGGTTAAATCCA | TGAAGAGCTTTGTCTACGCGA |
| *Ifnb1* | CGTGGGAGATGTCCTCAACT | CCTGAAGATCTCTGCTCGGAC |
| *Ifnb2* | CAGCTCCAAGAAAGGACGAAC | GGCAGTGTAACTCTTCTGCAT |
| *Isg15* | CGATTTCCTGGTGTCCGTGA | CTCGCTGCAGTTCTGTACCA |
| *Isg20* | GCTACACAAGAACATCCAGAACAAC | TCTGAGTCGCTGAGAGATTTTGT |
